# Extracellular Leakage Protein Patterns in Two Types of Cancer Cell Death: Necrosis and Apoptosis

**DOI:** 10.1021/acsomega.3c01691

**Published:** 2023-07-07

**Authors:** Akira Sato, Akira Shimotsuma, Tetsuya Miyoshi, Yui Takahashi, Naoki Funayama, Yoko Ogino, Akiko Hiramoto, Yusuke Wataya, Hye-Sook Kim

**Affiliations:** † Department of Biochemistry and Molecular Biology, Faculty of Pharmaceutical Sciences, Tokyo University of Science, Noda, Chiba 278-8510, Japan; ‡ Department of Gene Regulation, Faculty of Pharmaceutical Sciences, Tokyo University of Science, Noda, Chiba 278-8510, Japan; § Division of International Infectious Diseases Control, Faculty of Pharmaceutical Sciences, Okayama University, 1-1-1 Tsushima-naka, Kita-ku, Okayama 700-8530, Japan

## Abstract

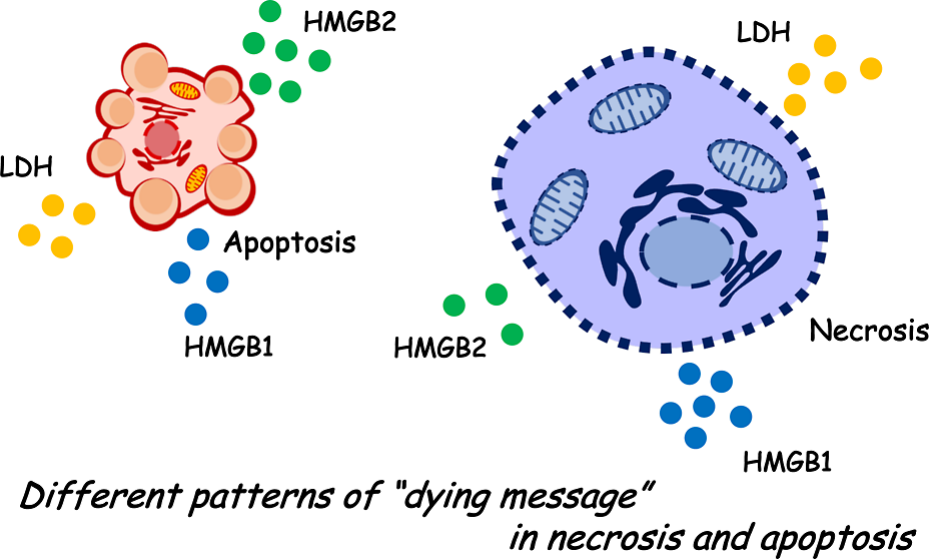

Dead cells release fragments of DNA, RNA, and proteins (including peptides) into the extracellular space. Two major forms of cell death during cancer development have been identified: necrosis and apoptosis. Our group investigated the mechanisms that regulate cell death during the treatment of mouse tumor FM3A cells with the anticancer drug floxuridine (FUdR). In the original strain F28-7, FUdR induced necrosis, whereas in the variant F28-7-A, it induced apoptosis. Here, we report that the extracellular leakage proteome (*i.e.*, the secretome) is involved in these cell death phenomena. The secretome profile, which was analyzed *via* shotgun proteomic analysis, revealed that altered protein leakage was involved in signal transduction, transcription, RNA processing, translation, and cell death. Notably, the characteristic secretory proteins high mobility group box 1 and 2 were detected in the culture medium of both necrotic and apoptotic cells. Overall, these results indicate that unique cellular events mediated by secretory proteins may be involved in necrosis and apoptosis.

## Introduction

Dead cells release fragments of DNA, RNA, and proteins (including peptides) into the extracellular space that function as damage-associated molecular patterns (DAMPs) and cytokines/chemokines.^1−3^ Two major forms of cancer cell death have been identified: necrosis (including programed necrosis) and apoptosis. DAMPs and cytokines/chemokines are released from both necrotic and apoptotic cells.^3−5^ Our group previously investigated the molecular mechanisms that regulate cell death during the treatment of mouse tumor FM3A cells with the anticancer drug floxuridine (5-fluoro-2′-deoxyuridine, FUdR) and showed that in the original strain F28-7, FUdR induces necrosis, while in the variant strain F28-7-A, it induces apoptosis.^6,7^ Necrosis in F28-7 is characterized by the swelling of cells and organelles and disruption of cellular and nuclear membranes^6,7^ and accompanied by cleavage of the apoptosis marker proteins caspase-3 and poly(ADP-ribose) polymerase-1 (PARP-1) and breakdown of DNA into chromosome-sized fragments.^6,7^ In contrast, apoptosis in F28-7-A is characterized by membrane blebbing, cell and organelle shrinkage, cytochrome *c* release from mitochondria, caspase-3 and PARP-1 cleavage, and oligonucleosomal DNA size fragmentation.^6−8^ Previously, we reported five possible regulators of necrosis and apoptosis: molecular chaperone heat shock protein 90 (HSP90),^8^ nuclear scaffold lamin-B1,^9,10^ cytoplasmic intermediate filament cytokeratin-19,^10^ transcription factor activating transcription factor 3 (ATF3),^11^ and microRNAs—miRNA-351-5p and miRNA-743a-3p.^12−14^ These cell death regulators were discovered by proteomic and transcriptomic analyses of dying cells using various approaches, including small interfering RNA, miRNA mimic, and miRNA inhibitor.^8−12^ In the present study, to understand the leakage protein patterns underlying necrosis and apoptosis, we investigated the extracellular whole protein profiles of necrosis in F28-7 cells and apoptosis in F28-7-A cells using a proteome analysis approach. This analysis revealed that the proteins involved in signal transduction, transcription, RNA processing, translation, and cell death presented altered leakage patterns during FUdR-induced necrosis and apoptosis. In addition, leakage of the cell death marker lactate dehydrogenase (LDH) occurred at higher levels in necrosis than in apoptosis. Interestingly, high mobility group box (HMGB) 1 and 2 were differentially leaked in both necrosis and apoptosis, respectively. Notably, the characteristic secretory protein galectin-3-binding protein was specifically detected in the culture medium of apoptotic cells. This study also discussed the possible regulatory mechanisms of the identified leakage proteins in necrosis and apoptosis.

## Materials and Methods

### Reagents

Floxyuridine (5-fluoro-2′-deoxyuridine, FUdR) was obtained from Sigma-Aldrich (Merck KGaA, Darmstadt, Germany). FUdR was stored in 2 mM stock solutions in ultrapure water at −20 °C.

### Cell Culture

The original F28-7 strain and the variant F28-7-A strain of mouse mammary carcinoma FM3A cells used in this study were described previously.^6,10^ FM3A cells were maintained in a suspension culture. The cells were grown at 37 °C under a humidified 5% CO_2_ atmosphere in ES medium containing 2% heat-inactivated fetal bovine serum (normal media). F28-7 and F28-7-A cells (approximately 5 × 10^5^ cells/mL) were treated with 1 μM FUdR. Cell viability was estimated using a hemocytometer based on trypan blue exclusion.

### Shotgun Proteomics of Extracellular Proteins

A proteomic secretome analysis was performed as previously described.^15,16^ Briefly, the cell culture medium was replaced with a serum-free ES medium. After treatment with 1 μM FUdR for 21 h, the cell culture medium of F28-7 or F28-7-A cells was centrifuged at 50*g* for 10 min, and then the supernatants were collected, condensed, and subjected to tryptic digestion. Label-free quantitative proteomic analysis of secretomes was performed *via* mass spectrometry by Medical ProteoScope Co., Ltd. (Yokohama, Japan).

### Detection of Extracellular LDH

Extracellular LDH activity was analyzed in the cell culture medium (with 2% FBS) using an LDH cytotoxicity detection kit (Takara Bio Inc., Shiga, Japan) according to the manufacturer’s instructions. The culture medium of F28-7 or F28-7-A cells was centrifuged at 50*g* for 10 min, and the supernatant was assayed using an LDH detection kit.

### Immunoblot Analysis

Condensation of extracellular proteins in the culture medium was performed using Amicon Ultra-2 centrifugal filters (2 mL capacity) using −30K and −100K devices (Merck KGaA), according to the manufacturer’s instructions. SDS-PAGE and immunoblot analysis were performed as previously described.^10,11^ The following antibodies were used: anti-HMGB1 rabbit monoclonal antibody (EPR3507, Abcam, Cambridge, UK), anti-HMGB2 (D1P9V) rabbit monoclonal antibody (9045S, 1:1000, Cell Signaling Technologies, Massachusetts, USA), and horseradish peroxidase-linked anti-rabbit IgG (1:20,000, GE Healthcare, Connecticut, USA).

### Statistical Analysis

Data are presented as the mean ± standard error of the mean. Significant differences between groups were determined by one-way analysis of variance (ANOVA) followed by Tukey’s multiple comparison test. *P* < 0.05 was considered significant. Statistical analyses were performed using GraphPad Prism 9 software.

## Results and Discussion

### Identification of the Extracellularly Leaked Proteins in Necrotic and Apoptotic Cell Culture Media

**Figure 1:**
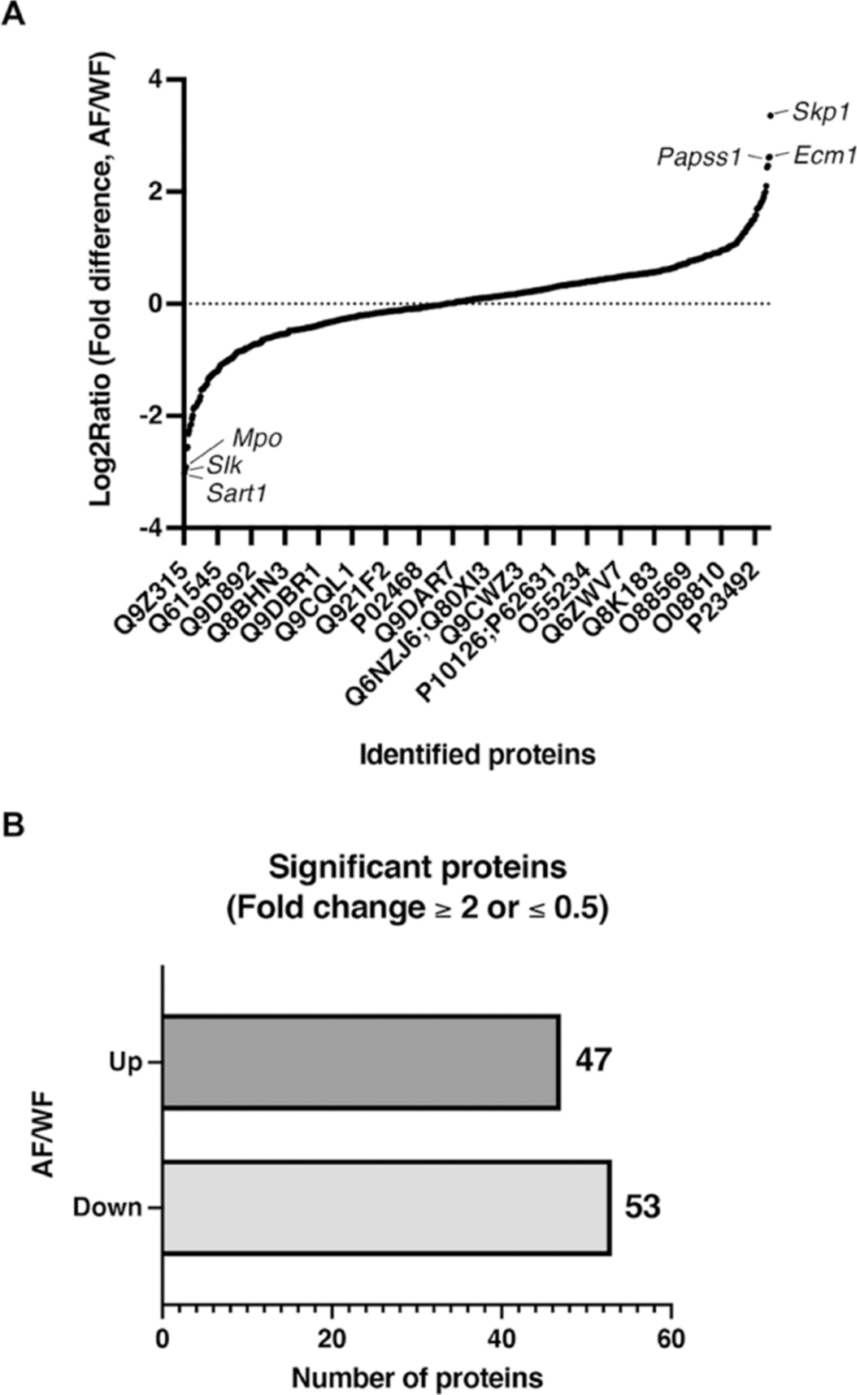
Identification of extracellularly leaked proteins in necrotic and apoptotic cell culture media. (A) Results of the proteomic analysis performed as a pilot study (single experiment). In total, 699 unique proteins were identified in necrotic and apoptotic cell culture media. (B) Significantly leaked proteins. In total, 53 proteins corresponded to the higher cutoff threshold (≥2) and 47 proteins corresponded to the lower threshold (0.5≥) in the apoptotic cell culture medium compared with the necrotic cell culture medium. AF indicates apoptotic cell culture medium. F28-7-A cells were treated with 1 μM FUdR for 21 h to induce apoptosis. WF indicates necrotic cell culture medium. F28-7 cells were treated with 1 μM FUdR for 21 h to induce necrosis.

We investigated the leaked protein patterns in necrotic and apoptotic cells using a shotgun proteomic approach. The F28-7 and F28-7-A cells were treated with 1 μM FUdR for 21 h, in which FUdR induced necrosis in F28-7 cells and apoptosis in F28-7-A cells. Under this condition, necrosis is predominant in F28-7 cells, while apoptosis is predominant in F28-7-A cells.^6,12^ Necrotic and apoptotic cell culture media were analyzed using a nano-LC–MS/MS system to detect changes in the necrotic and apoptotic leakage proteomes (secretomes). Figure 1A and Table S1 summarize the 699 extracellularly leaked proteins identified in the necrotic and apoptotic cell culture media. Based on a 2.0-fold cutoff of either ≥2.0 or 0.5≥, the analysis identified 100 altered leaked proteins between apoptosis and necrosis, with 47 leaked proteins ≥2.0 and 53 leaked proteins 0.5≥ (Figure 1B). Tables 1 and 2 present the top 10 upregulated and downregulated proteins in the apoptotic cell culture medium compared to those in the necrotic cell culture medium, respectively. These proteins may simply be necrotic or apoptotic death messages to the surrounding healthy cells or cell death signals that transmit information leading to cell death. Notably, our previous proteome data indicated that the intracellular protein level of histone H2A type 3 was 3.3-fold higher in FUdR-treated F28-7 cells (necrosis cells) than in FUdR-treated F28-7-A cells (apoptosis).^10^ However, in our present secretome data, the extracellular leakage level of histone H2A type 3 was 5.4-fold higher in FUdR-treated F28-7-A cells (apoptosis) than in FUdR-treated F28-7 cells (necrosis cells) (Table 1). These findings suggest that histone H2A type 3 is extracellularly leaked during the apoptosis processes. Interestingly, the protein galectin-3-binding protein (LGALS3BP) was only identified in the apoptotic cell culture medium (Table 3). LGALS3BP is involved in cancer progression and metastasis;^17^ however, its involvement in regulating cell death is not well understood. In the future, we intend to investigate the role of this molecule in necrotic and apoptotic cell death machinery. Importantly, these results indicate that unique cellular events mediated by secretory proteins may be involved in necrosis and apoptosis.

**Table 1:** Top 10 Upregulated Proteins in the Apoptotic Culture Medium Compared to Those in the Necrotic Culture Medium^a^

accession no.	description (*gene symbol*)	ratio (AF/WF)	biological process	cellular component
Q9WTX5	S-phase kinase-associated protein 1 (*Skp1*)	10.22	Ubl conjugation pathway	cytoplasm/extracellular exosome
Q61508	extracellular matrix protein 1 (*Ecm1*)	6.13	mineral balance	secreted
Q60967	bifunctional 3′-phosphoadenosine 5′-phosphosulfate synthase 1 (*Papss1*)	6.10	sulfate assimilation	sulfate adenylyltransferase complex
Q8BFU2	histone H2A type 3 (*Hist3h2a*)	5.39	chromatin organization	nucleus
P26043	radixin (*Rdx*)	4.29	actin capping	cytoplasm/membrane
Q8BFS6	serine/threonine-protein phosphatase CPPED1 (*Cpped1*)	3.99	protein modification	cytoplasm
P62082	40S ribosomal protein S7 (*Rps7*)	3.92	translation	cytoplasm
Q3U0V1	far upstream element-binding protein 2 (*Khsrp*)	3.73	transcription	nucleus/cytoplasm
P51855	glutathione synthetase (*Gss*)	3.63	glutathione biosynthesis	extracellular exosome
Q810D6	glutamate-rich WD repeat-containing protein 1 (*Grwd1*)	3.54	unknown	nucleus

a Accession no. corresponds to the UniProt Knowledgebase accession number. The column “description” indicates the identified protein name and gene symbol in *Mus musculus*.

**Table 2:** Top 10 Downregulated Proteins in the Apoptotic Culture Medium Compared to Those in the Necrotic Culture Medium^a^

accession no.	description (*gene symbol*)	ratio (AF/WF)	biological process	cellular component
Q9Z315	U4/U6.U5 tri-snRNP-associated protein 1 (*Sart1*)	0.12	mRNA processing	nucleus
O54988	STE20-like serine/threonine-protein kinase (*Slk*)	0.13	apoptosis	cytoplasm
P11247	myeloperoxidase (*Mpo*)	0.17	hydrogen peroxide	lysosome
P62996	transformer-2 protein homolog β (*Tra2b*)	0.17	mRNA processing	nucleus
Q9JHU9	inositol-3-phosphate synthase 1 (*Isyna1*)	0.20	biosynthesis	cytoplasm
E9Q401	ryanodine receptor 2 (*Ryr2*)	0.21	calcium transport	membrane
Q3V0B4	coiled-coil domain-containing protein 108 (*Ccdc108*)	0.22	unknown	membrane
P48024	eukaryotic translation initiation factor 1 (*Eif1*)	0.23	protein biosynthesis	nucleus/cytoplasm
Q9DBG3	AP-2 complex subunit β (*Ap2b1*)	0.24	protein transport	membrane
Q62376	U1 small nuclear ribonucleoprotein 70 kDa (*Snrnp70*)	0.25	mRNA splicing	nucleus

a Accession no. corresponds to the UniProt Knowledgebase accession number. The column “description” indicates the identified protein name and gene symbol in *M. musculus*.

**Table 3:** Specific Protein Identified in the Apoptotic Culture Medium^a^

accession no.	description (*gene symbol*)	biological process	cellular component
Q07797	**galectin-3-bindingprotein (*Lgals3bp*)**	cell adhesion	secreted

a Accession no. corresponds to the UniProt Knowledgebase accession number. The column “description” indicates the identified protein name and gene symbol in *M. musculus*.

### Biological Classification of the Identified Extracellularly Leaked Proteins in Necrotic and Apoptotic Cell Culture Media

**Figure 2:**
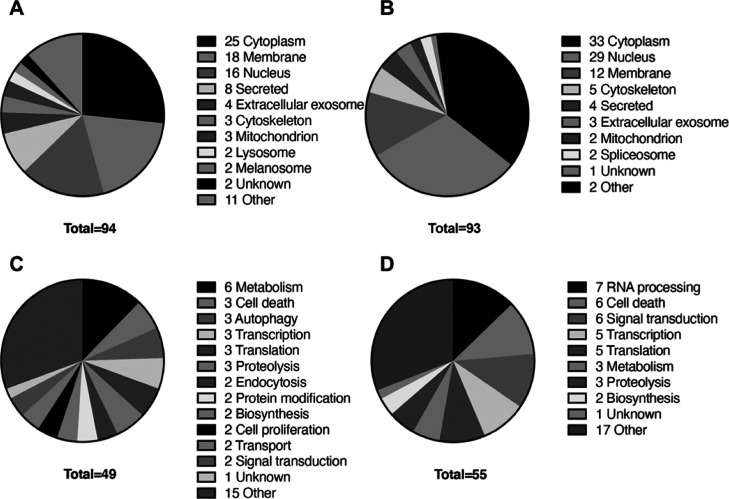
Biological classification of the identified leaked proteins in necrotic and apoptotic culture media. (A,B) Cellular component classification of the identified proteins. (A) Increased proteins in apoptotic cell culture medium. (B) Decreased proteins in apoptotic cell culture medium. (C,D) Biological process classification of the identified proteins. (C) Increased proteins in apoptotic cell culture medium. (D) Decreased proteins in apoptotic cell culture medium.

Next, we analyzed the cellular components and biological processes associated with alterations in the extracellularly leaked proteins in the necrotic and apoptotic cell culture media. Protein localization differed between necrosis and apoptosis, resulting in diverse localizations, including the cytoplasm, nucleus, membrane, cytoskeleton, and mitochondria (Figure 2A,B). Nuclear proteins, such as histone H2A type 3, glutamate-rich WD repeat-containing protein 1, U4/U6. U5 tri-snRNP-associated protein 1, transformer-2 protein homolog β, and U1 small nuclear ribonucleoprotein 70 kDa, presented different leakage patterns during necrosis and apoptosis (Tables 1 and 2). The biological processes and functions associated with these leakage proteins include RNA processing, cell death, signal transduction, metabolism, transcription, and translation (Figure 2C,D). These findings indicate that proteins involved in various cellular localizations and biological processes are leaked and/or secreted during necrosis and apoptosis.

### Extracellular Leakage Patterns of Cell Death Hallmark Proteins LDH and HMGB Proteins in Necrotic and Apoptotic Cell Culture Media

**Figure 3:**
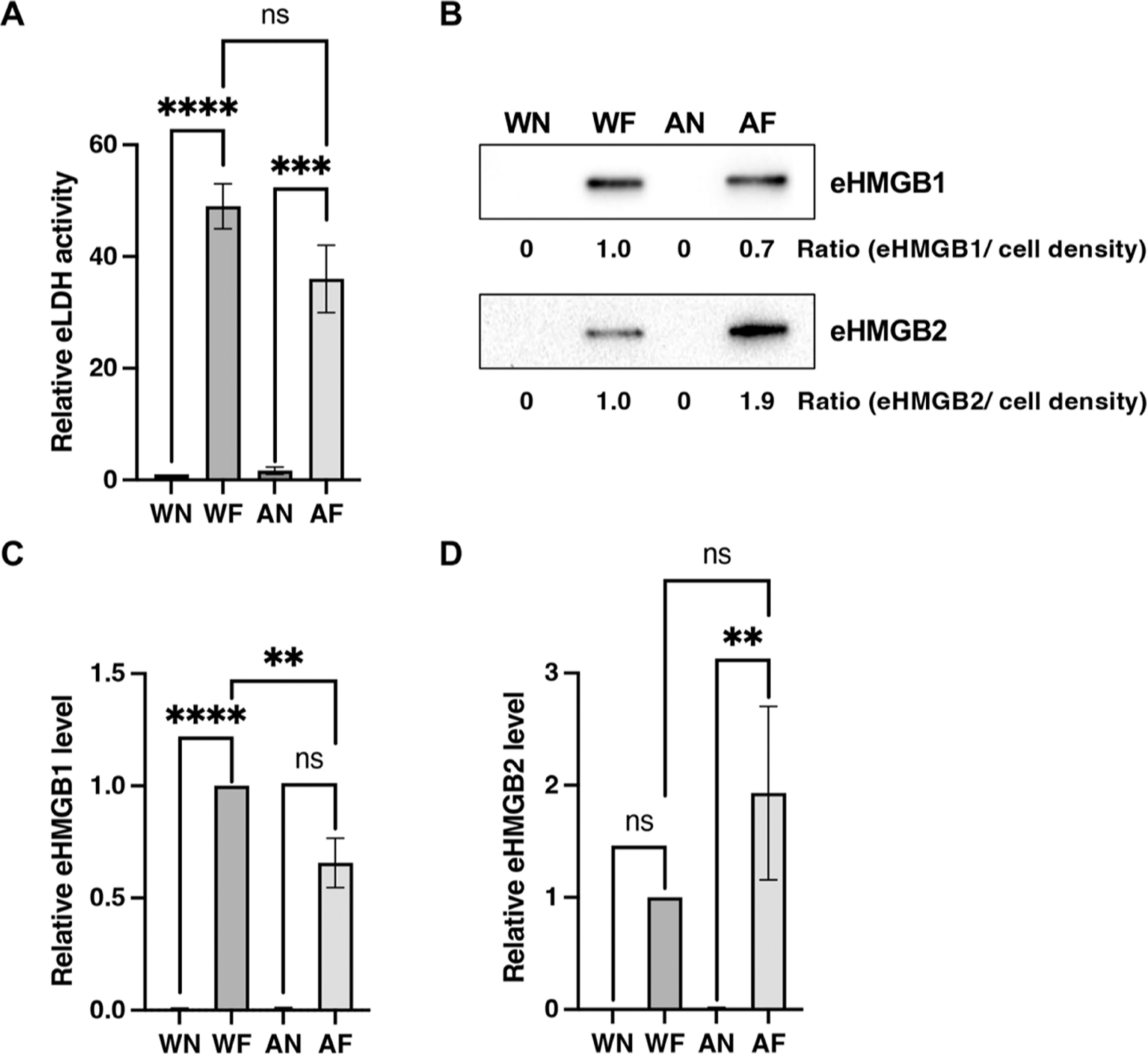
Leakage patterns of the cell death hallmark proteins lactose dehydrogenase and HMGB in necrotic and apoptotic culture media. (A) Relative extracellular LDH activity determined by an LDH cytotoxicity detection kit. F28-7 cells and F28-7-A cells were treated with or without 1 μM FUdR for 21 h. eLDH, extracellular LDH. WN, untreated F28-7 cells. WF, FUdR-treated F28-7 cells. AN, untreated F28-7-A cells. AF, FUdR-treated F28-7-A cells. Relative values for each LDH activity are shown as 1 for the WN group. Results are the average of three independent experiments, with error bars indicating ±SE. One-way ANOVA followed by Tukey’s multiple comparison test: ****, *p* < 0.0001 (WF *vs* WN) and ***, *p* < 0.001 (AF *vs* AN). ns, not significant (AF *vs* WF). (B) Western blot image of extracellular HMGB1 and HMGB2. The HMGB1 and HMGB2 protein levels were examined by immunoblotting. F28-7 cells and F28-7-A cells were treated with or without 1 μM FUdR for 21 h. The ratio shows the intensity of each protein band corrected for each cell density. Data are representative of at least three independent experiments. eHMGB1, extracellular HMGB1; eHMGB2, extracellular HMGB2. (C) Relative extracellular HMGB1 level. Relative values for each HMGB1 levels are shown as 1 for the WF group. Results are the average of three independent experiments, with error bars indicating ±SE. One-way ANOVA followed by Tukey’s multiple comparison test: ****, *p* < 0.0001 (WF *vs* WN and AF *vs* AN) and ***, *p* < 0.001 (AF *vs* WF). (D) Relative extracellular HMGB2 level. Relative values for each HMGB2 levels are shown as 1 for the WF group. Results are the averages of three independent experiments with error bars indicating ±SE. One-way ANOVA followed by Tukey’s multiple comparison test: ns, not significant (WF *vs* WN). **, *p* < 0.01 (AF *vs* AN). ns, not significant (AF *vs* WF).

Previous studies have shown that LDH and HMGB1 leak from dead and damaged cells.^18−22^ In particular, HMGB1, which is one of the most extensively studied DAMPs,^3,23^ represents a cell death marker known to leak from necrotic and apoptotic cells.^19,24^ Indeed, proteome analysis results identified LDH, HMGB1, and related proteins HMGB2 and HMGB3 from necrotic and apoptotic cell culture media (Table 4). Extracellular LDH activity was lower in the apoptotic cell culture medium than in the necrotic cell culture medium, while extracellular HMGB1 levels were similar in the necrotic and apoptotic cell culture media. Of note, HMGB2 and HMGB3 levels were higher in the apoptotic medium than in the necrotic cell culture medium. To validate these proteomic data, LDH and HMGB1/2 were quantified by LDH cytotoxicity detection assay and immunoblotting, respectively. The assay measures the activity of two LDH isozymes, LDHA and LDHB, in cell culture medium and showed that extracellular LDH activity was 0.7-fold lower in the apoptotic cell culture medium than in the necrotic cell culture medium (Figure 3A). In addition, extracellular HMGB1 levels were 0.7-fold lower in the apoptotic necrotic cell culture medium than in the necrotic cell culture medium (Figure 3B,C) while extracellular HMGB2 levels were 1.9-fold higher in the apoptotic necrotic cell culture medium than in the necrotic cell culture medium (Figure 3B,D). These differences in LDH activity and HMGB2 levels were generally consistent with the proteomic analysis. In contrast, the difference in the HMGB1 level was inconsistent with the proteomic data. Importantly, LDH is released from damaged cells, and this study revealed that it leaked from both necrotic and apoptotic cells; moreover, HMGB1 and HMGB2 were also released from both necrotic and apoptotic cells. The HMGB family includes four members: HMGB1, 2, 3, and 4.^23,25^ HMGB1, 2, and 3 share more than 80% identity at the amino acid sequence level and have similar biochemical properties. These proteins are composed of two DNA-binding HMG domains and an acidic tail.^23,26^ HMGB proteins have two primary functions. In the nucleus, HMGB proteins bind to DNA in a DNA structure-dependent but nucleotide sequence-independent manner to function in chromatin remodeling.^23^ Outside the cell, HMGB proteins function as alarmins, which are endogenous molecules released upon tissue damage that activate the immune system.^23^ Notably, HMGB2 showed greater leakage from apoptotic cells than from necrotic cells. These data suggest that the leakage patterns of multiple molecules, such as LDH and HMGB1/2, may indicate different modes of cell death. Thus, future studies should perform detailed investigations on whether the leakage pattern of these proteins is due to the difference in cellular membrane conditions between necrotic cells and apoptotic cells or whether it is a specific leakage pattern associated with the difference in the cell death process between necrotic and apoptotic cells.

**Table 4:** Differential Leakage Patterns of LDH and HMGB Proteins in the Proteomics Data^a^

accession no.	description (*gene symbol*)	ratio (AF/WF)
P06151	l-lactate dehydrogenase A chain (*Ldha*)	0.59
P63158	high mobility group protein B1 (*Hmgb1*)	1.04
P30681	high mobility group protein B2 (*Hmgb2*)	1.45
O54879	high mobility group protein B3 (*Hmgb3*)	1.20

a Accession no. corresponds to the UniProt Knowledgebase accession number. The column “description” indicates the identified protein name and gene symbol in *M. musculus*.

Many studies have indicated that DAMPs from dying cells and stress trigger acute/chronic inflammation, thereby promoting the development or progression of tumors.^27,28^ In addition, the involvement of DAMPs in controlling excessive inflammation, resolving chronic inflammation, and promoting tissue repair and healing has been reported.^3^ Interestingly, Karsch-Bluman *et al.* reported that the secretion of necrotic factors during tumor necrosis can promote cancer progression by supporting different steps in the cascade of cancer formation and may result in resistance to treatment and tumor evasion.^29^ Furthermore, Wickman *et al.* reported that the formation of blebs and apoptotic bodies by actin–myosin contraction during apoptosis causes acute and localized release of multiple DAMPs, such as immunomodulatory proteins, before secondary necrosis occurs.^22^ This report suggests that the shift from apoptosis to secondary necrosis is more graded than a simple binary switch, with the membrane permeabilization of apoptotic bodies and the consequent limited release of DAMPs contributing to the transition between these states.^22^ These findings and our report suggest that DAMPs from necrotic and apoptotic cells may act as key players in the transmission of cell death modes and transduction of cell death signals.

One limitation of our present study is that a portion of the FUdR-treated F28-7-A cells undergoing apoptosis may have transitioned to secondary necrosis. Our future studies will focus on the differences in extracellular leakage molecules, DNA, RNA, and proteins in the early stage of necrosis and apoptosis.

## Conclusions

The present study shows that a wide variety of proteins are released from necrotic and apoptotic cells, and they may represent death signaling molecules or simply death messages to healthy cells. These findings may reveal novel cell death markers for determining the mode of cell death. Our research also shows that to classify cell death modes by cell death markers, multiple leakage proteins that are either increased or decreased during necrosis and apoptosis must be determined. In addition, our findings are important for exploring the roles of messages from dead cells in necrosis–apoptosis cell-death processes.

## Supporting Information Available

Identification of extracellular proteins in necrosis and apoptosis (PDF)

## Abbreviations

ATF3: activating transcription factor 3
DAMP: damage-associated molecular pattern
FUdR: floxuridine
HMGB: high mobility group box
LDH: lactose dehydrogenase
LGALS3BP: galectin-3-binding protein
PARP-1: poly(ADP-ribose) polymerase-1
