# Supplementary material for: Extracellular Leakage Protein Patterns in Two Types of Cancer Cell Death: Necrosis and Apoptosis
Source: ACS Omega. 2023 Jul 7;8(28):25059–65. doi: 10.1021/acsomega.3c01691 (PMC10357420; doi:10.1021/acsomega.3c01691)
Supplement: Supplementary file 1 — ao3c01691_si_001.pdf [file ao3c01691_si_001.pdf]

# Extracellular leakage protein patterns in two types of cancer cell death: necrosis and apoptosis

*Akira Sato[1,*], Akira Shimotsuma[1], Tetsuya Miyoshi[1], Yui Takahashi[1], Naoki Funayama[1], Yoko Ogino[1,2], Akiko Hiramoto[3], Yusuke Wataya[3], Hye-Sook Kim[3]*

[1] Department of Biochemistry and Molecular Biology, Faculty of Pharmaceutical Sciences, Tokyo University of Science, Noda, Chiba 278-8510, Japan.

[2] Department of Gene Regulation, Faculty of Pharmaceutical Sciences, Tokyo University of Science, Noda, Chiba 278-8510, Japan.

[3] Division of International Infectious Disease Control, Faculty of Pharmaceutical Sciences, Okayama University, 1-1-1 Tsushima-naka, Kita-ku, Okayama 700-8530, Japan.

**This document contain:**

**Table S1**: Identification of extracellular proteins in necrosis and apoptosis.

Table S1. Identification of extracellular proteins in necrosis and apoptosis

| Accession | Description | AF | WF | Ratio(AF/WF) |
|---|---|---|---|---|
| Q9Z315 | **U4/U6.U5 tri-snRNP-associated protein 1 OS=Mus musculus GN=Sart1 PE=2 SV=1** | 1148.4 | 9322.3 | 0.12 |
| O54988 | **STE20-like serine/threonine-protein kinase OS=Mus musculus GN=Slk PE=1 SV=2** | 1222.5 | 9466.0 | 0.13 |
| P00761 | **Contaminant (Trypsin)** | 147007.9 | 1104681.4 | 0.13 |
| P11247 | **Myeloperoxidase OS=Mus musculus GN=Mpo PE=2 SV=2** | 106654.1 | 637359.2 | 0.17 |
| P62996 | **Transformer-2 protein homolog beta OS=Mus musculus GN=Tra2b PE=2 SV=1** | 5909.1 | 34805.4 | 0.17 |
| Q9JHU9 | **Inositol-3-phosphate synthase 1 OS=Mus musculus GN=Isyna1 PE=2 SV=1** | 2113.1 | 10553.9 | 0.20 |
| E9Q401 | **Ryanodine receptor 2 OS=Mus musculus GN=Ryr2 PE=1 SV=1** | 3546.9 | 16972.0 | 0.21 |
| Q3V0B4 | **Coiled-coil domain-containing protein 108 OS=Mus musculus GN=Ccdc108 PE=2 SV=1** | 1228.5 | 5567.3 | 0.22 |
| P48024 | **Eukaryotic translation initiation factor 1 OS=Mus musculus GN=Eif1 PE=3 SV=2** | 2464.7 | 10929.9 | 0.23 |
| Q9DBG3 | **AP-2 complex subunit beta OS=Mus musculus GN=Ap2b1 PE=1 SV=1** | 4072.8 | 16840.7 | 0.24 |
| Q62376 | **U1 small nuclear ribonucleoprotein 70 kDa OS=Mus musculus GN=Snrnp70 PE=1 SV=2** | 4839.1 | 19217.6 | 0.25 |
| P02798 | **Metallothionein-2 OS=Mus musculus GN=Mt2 PE=1 SV=2** | 1303.4 | 4781.5 | 0.27 |
| P29699 | **Alpha-2-HS-glycoprotein OS=Mus musculus GN=Ahsg PE=1 SV=1** | 96687.7 | 346986.9 | 0.28 |
| P05784 | **Keratin, type I cytoskeletal 18 OS=Mus musculus GN=Krt18 PE=1 SV=5** | 83724.9 | 299164.1 | 0.28 |
| P11679 | **Keratin, type II cytoskeletal 8 OS=Mus musculus GN=Krt8 PE=1 SV=4** | 50350.8 | 178632.9 | 0.28 |
| P46061 | **Ran GTPase-activating protein 1 OS=Mus musculus GN=Rangap1 PE=1 SV=2** | 2179.4 | 7596.6 | 0.29 |
| Q9CQJ6 | **Density-regulated protein OS=Mus musculus GN=Denr PE=2 SV=1** | 13323.3 | 45275.0 | 0.29 |
| P10648 | **Glutathione S-transferase A2 OS=Mus musculus GN=Gsta2 PE=1 SV=3** | 96923.2 | 324008.8 | 0.30 |
| Q62348 | **Translin OS=Mus musculus GN=Tsn PE=1 SV=1** | 2585.2 | 8499.4 | 0.30 |
| P43276 | **Histone H1.5 OS=Mus musculus GN=Hist1h1b PE=1 SV=2** | 11977.5 | 37720.7 | 0.32 |
| Q9CYZ2 | **Tumor protein D54 OS=Mus musculus GN=Tpd52l2 PE=1 SV=1** | 29285.6 | 85087.2 | 0.34 |
| P62869 | **Transcription elongation factor B polypeptide 2 OS=Mus musculus GN=Tceb2 PE=1 SV=1** | 20642.8 | 59344.0 | 0.35 |
| O08529 | **Calpain-2 catalytic subunit OS=Mus musculus GN=Capn2 PE=2 SV=4** | 11134.8 | 31966.6 | 0.35 |
| P97494 | **Glutamate--cysteine ligase catalytic subunit OS=Mus musculus GN=Gclc PE=2 SV=4** | 1486.3 | 4243.0 | 0.35 |
| Q9JJA4 | **Ribosome biogenesis protein WDR12 OS=Mus musculus GN=Wdr12 PE=2 SV=1** | 7301.6 | 20394.0 | 0.36 |
| Q8VHX6 | **Filamin-C OS=Mus musculus GN=Flnc PE=1 SV=3** | 85685.7 | 235813.3 | 0.36 |
| Q9CXW3 | **Calcyclin-binding protein OS=Mus musculus GN=Cacybp PE=1 SV=1** | 48253.7 | 131906.2 | 0.37 |
| O70400 | **PDZ and LIM domain protein 1 OS=Mus musculus GN=Pdlim1 PE=2 SV=4** | 103217.9 | 277064.3 | 0.37 |
| Q8K4Z5 | **Splicing factor 3A subunit 1 OS=Mus musculus GN=Sf3a1 PE=1 SV=1** | 11509.0 | 29287.7 | 0.39 |
| E9Q414 | **Apolipoprotein B-100 OS=Mus musculus GN=Apob PE=1 SV=1** | 74485.9 | 186582.3 | 0.40 |
| Q69ZN7 | **Myoferlin OS=Mus musculus GN=Myof PE=1 SV=2** | 28347.1 | 70835.7 | 0.40 |

| | | | | |
|---|---|---|---|---|
| O54962 | **Barrier-to-autointegration factor OS=Mus musculus GN=Banf1 PE=1 SV=1** | 3973.8 | 9822.9 | 0.40 |
| P83940 | **Transcription elongation factor B polypeptide 1 OS=Mus musculus GN=Tceb1 PE=1 SV=1** | 10063.0 | 24524.0 | 0.41 |
| P10639 | **Thioredoxin OS=Mus musculus GN=Txn PE=1 SV=3** | 386428.2 | 932849.4 | 0.41 |
| P61089 | **Ubiquitin-conjugating enzyme E2 N OS=Mus musculus GN=Ube2n PE=1 SV=1** | 44993.3 | 107000.1 | 0.42 |
| Q62084 | **Protein phosphatase 1 regulatory subunit 14B OS=Mus musculus GN=Ppp1r14b PE=1 SV=2** | 49133.1 | 116534.0 | 0.42 |
| P16546 | **Spectrin alpha chain, non-erythrocytic 1 OS=Mus musculus GN=Sptan1 PE=1 SV=4** | 89097.5 | 207678.4 | 0.43 |
| P14069 | **Protein S100-A6 OS=Mus musculus GN=S100a6 PE=1 SV=3** | 162896.6 | 378516.7 | 0.43 |
| Q9Z2W1 | **Serine/threonine-protein kinase 25 OS=Mus musculus GN=Stk25 PE=2 SV=2** | 3371.0 | 7832.6 | 0.43 |
| Q6PDM2 | **Serine/arginine-rich splicing factor 1 OS=Mus musculus GN=Srsf1 PE=1 SV=3** | 105434.4 | 243204.6 | 0.43 |
| Q61545 | **RNA-binding protein EWS OS=Mus musculus GN=Ewsr1 PE=1 SV=2** | 24916.2 | 56926.0 | 0.44 |
| Q9CZY3 | **Ubiquitin-conjugating enzyme E2 variant 1 OS=Mus musculus GN=Ube2v1 PE=1 SV=1** | 7746.4 | 17312.5 | 0.45 |
| P68033 | **Actin, alpha cardiac muscle 1 OS=Mus musculus GN=Actc1 PE=1 SV=1** | 415632.0 | 911091.6 | 0.46 |
| P84104 | **Serine/arginine-rich splicing factor 3 OS=Mus musculus GN=Srsf3 PE=1 SV=1** | 132350.3 | 283411.5 | 0.47 |
| Q8QZY1 | **Eukaryotic translation initiation factor 3 subunit L OS=Mus musculus GN=Eif3l PE=1 SV=1** | 60604.8 | 128159.4 | 0.47 |
| Q9CQV8 | **14-3-3 protein beta/alpha OS=Mus musculus GN=Ywhab PE=1 SV=3** | 359572.3 | 758173.0 | 0.47 |
| P15532 | **Nucleoside diphosphate kinase A OS=Mus musculus GN=Nme1 PE=1 SV=1** | 10235.5 | 21420.1 | 0.48 |
| Q99JF8 | **PC4 and SFRS1-interacting protein OS=Mus musculus GN=Psip1 PE=1 SV=1** | 33036.6 | 68976.6 | 0.48 |
| P62900 | **60S ribosomal protein L31 OS=Mus musculus GN=Rpl31 PE=1 SV=1** | 38320.3 | 79051.4 | 0.48 |
| P05063 | **Fructose-bisphosphate aldolase C OS=Mus musculus GN=Aldoc PE=1 SV=4** | 7071.0 | 14560.7 | 0.49 |
| P62204 | **Calmodulin OS=Mus musculus GN=Calm1 PE=1 SV=2** | 1210615.1 | 2475376.8 | 0.49 |
| Q8WTY4 | **Anamorsin OS=Mus musculus GN=Ciapin1 PE=1 SV=1** | 35335.9 | 71887.3 | 0.49 |
| Q61398 | **Procollagen C-endopeptidase enhancer 1 OS=Mus musculus GN=Pcolce PE=1 SV=2** | 88403.3 | 177805.1 | 0.50 |
| P26350 | **Prothymosin alpha OS=Mus musculus GN=Ptma PE=1 SV=2** | 4109.7 | 8209.8 | 0.50 |
| P25206 | **DNA replication licensing factor MCM3 OS=Mus musculus GN=Mcm3 PE=1 SV=2** | 8491.5 | 16803.4 | 0.51 |
| Q8BJU0 | **Small glutamine-rich tetratricopeptide repeat-containing protein alpha OS=Mus musculus GN=Sgta PE=1 SV=2** | 1687.1 | 3328.4 | 0.51 |
| P12023 | **Amyloid beta A4 protein OS=Mus musculus GN=App PE=1 SV=3** | 12462.7 | 24490.5 | 0.51 |
| P97855 | **Ras GTPase-activating protein-binding protein 1 OS=Mus musculus GN=G3bp1 PE=1 SV=1** | 48118.4 | 94361.7 | 0.51 |
| Q3UZ39 | **Leucine-rich repeat flightless-interacting protein 1 OS=Mus musculus GN=Lrrfip1 PE=1 SV=2** | 5811.7 | 11144.3 | 0.52 |
| Q9DBD0 | **Inhibitor of carbonic anhydrase OS=Mus musculus GN=Ica PE=1 SV=1** | 33620.3 | 64434.0 | 0.52 |
| Q99JR5 | **Tubulointerstitial nephritis antigen-like OS=Mus musculus GN=Tinagl1 PE=1 SV=1** | 25510.2 | 47984.8 | 0.53 |

| | | | | |
|---|---|---|---|---|
| P11031 | **Activated RNA polymerase II transcriptional coactivator p15 OS=Mus musculus GN=Sub1 PE=1 SV=3** | 58105.7 | 107922.5 | 0.54 |
| Q3UHX2 | **28 kDa heat- and acid-stable phosphoprotein OS=Mus musculus GN=Pdap1 PE=1 SV=1** | 122105.8 | 225691.1 | 0.54 |
| Q9D8S4 | **Oligoribonuclease, mitochondrial OS=Mus musculus GN=Rexo2 PE=1 SV=2** | 27852.6 | 50705.4 | 0.55 |
| Q9D1P4 | **Cysteine and histidine-rich domain-containing protein 1 OS=Mus musculus GN=Chordc1 PE=1 SV=1** | 13958.5 | 25371.1 | 0.55 |
| O08749 | **Dihydrolipoyl dehydrogenase, mitochondrial OS=Mus musculus GN=Dld PE=1 SV=2** | 24444.0 | 44105.1 | 0.55 |
| Q61686 | **Chromobox protein homolog 5 OS=Mus musculus GN=Cbx5 PE=1 SV=1** | 7409.9 | 13340.6 | 0.56 |
| O70251 | **Elongation factor 1-beta OS=Mus musculus GN=Eef1b PE=1 SV=5** | 698462.6 | 1255445.7 | 0.56 |
| P05201 | **Aspartate aminotransferase, cytoplasmic OS=Mus musculus GN=Got1 PE=1 SV=3** | 101864.7 | 182354.3 | 0.56 |
| P18760 | **Cofilin-1 OS=Mus musculus GN=Cfl1 PE=1 SV=3** | 1229495.6 | 2189289.4 | 0.56 |
| Q9QXS1 | **Plectin OS=Mus musculus GN=Plec PE=1 SV=3** | 147296.8 | 262247.5 | 0.56 |
| P37889 | **Fibulin-2 OS=Mus musculus GN=Fbln2 PE=1 SV=2** | 50437.5 | 89041.2 | 0.57 |
| Q61656 | **Probable ATP-dependent RNA helicase DDX5 OS=Mus musculus GN=Ddx5 PE=1 SV=2** | 30403.6 | 53570.0 | 0.57 |
| Q99L47 | **Hsc70-interacting protein OS=Mus musculus GN=St13 PE=1 SV=1** | 123776.8 | 214577.3 | 0.58 |
| P47753 | **F-actin-capping protein subunit alpha-1 OS=Mus musculus GN=Capza1 PE=1 SV=4** | 5414.3 | 9382.1 | 0.58 |
| Q9WVA4 | **Transgelin-2 OS=Mus musculus GN=Tagln2 PE=1 SV=4** | 726308.7 | 1254867.3 | 0.58 |
| Q920E5 | **Farnesyl pyrophosphate synthase OS=Mus musculus GN=Fdps PE=1 SV=1** | 351894.6 | 604912.0 | 0.58 |
| Q9WU28 | **Prefoldin subunit 5 OS=Mus musculus GN=Pfdn5 PE=2 SV=1** | 18352.1 | 31475.0 | 0.58 |
| P06151 | **L-lactate dehydrogenase A chain OS=Mus musculus GN=Ldha PE=1 SV=3** | 1754016.9 | 2973974.3 | 0.59 |
| Q8BKC5 | **Importin-5 OS=Mus musculus GN=Ipo5 PE=1 SV=3** | 38187.7 | 64571.6 | 0.59 |
| Q9D892 | **Inosine triphosphate pyrophosphatase OS=Mus musculus GN=Itpa PE=1 SV=2** | 23093.3 | 38810.1 | 0.60 |
| P61358 | **60S ribosomal protein L27 OS=Mus musculus GN=Rpl27 PE=1 SV=2** | 117201.1 | 195719.3 | 0.60 |
| P57776 | **Elongation factor 1-delta OS=Mus musculus GN=Eef1d PE=1 SV=3** | 220844.1 | 367347.3 | 0.60 |
| Q60972 | **Histone-binding protein RBBP4 OS=Mus musculus GN=Rbbp4 PE=1 SV=5** | 41496.1 | 68723.5 | 0.60 |
| P68368 | **Tubulin alpha-4A chain OS=Mus musculus GN=Tuba4a PE=1 SV=1** | 21651.3 | 35664.7 | 0.61 |
| Q9Z1B5 | **Mitotic spindle assembly checkpoint protein MAD2A OS=Mus musculus GN=Mad2l1 PE=1 SV=2** | 16848.4 | 27747.4 | 0.61 |
| P50247 | **Adenosylhomocysteinase OS=Mus musculus GN=Ahcy PE=1 SV=3** | 181636.3 | 298787.4 | 0.61 |
| P02769 | **Contaminant (Serum albumin)** | 72528327.1 | 119256341.1 | 0.61 |
| Q5XJY5 | **Coatomer subunit delta OS=Mus musculus GN=Arcn1 PE=1 SV=2** | 5901.0 | 9659.9 | 0.61 |
| P63005 | **Platelet-activating factor acetylhydrolase IB subunit alpha OS=Mus musculus GN=Pafah1b1 PE=1 SV=2** | 42726.5 | 69623.9 | 0.61 |
| Q9CQI3 | **Glia maturation factor beta OS=Mus musculus GN=Gmfb PE=1 SV=3** | 135177.3 | 219859.8 | 0.61 |
| Q8R317 | **Ubiquilin-1 OS=Mus musculus GN=Ubqln1 PE=1 SV=1** | 19067.7 | 30652.2 | 0.62 |
| P70195 | **Proteasome subunit beta type-7 OS=Mus musculus GN=Psmb7 PE=1 SV=1** | 54640.4 | 87660.6 | 0.62 |

| | | | | |
|---|---|---|---|---|
| Q61699 | **Heat shock protein 105 kDa OS=Mus musculus GN=Hsph1 PE=1 SV=2** | 74055.9 | 115348.6 | 0.64 |
| Q60864 | **Stress-induced-phosphoprotein 1 OS=Mus musculus GN=Stip1 PE=1 SV=1** | 412782.8 | 642018.3 | 0.64 |
| Q62048 | **Astrocytic phosphoprotein PEA-15 OS=Mus musculus GN=Pea15 PE=1 SV=1** | 41488.3 | 64509.5 | 0.64 |
| Q9D8N0 | **Elongation factor 1-gamma OS=Mus musculus GN=Eef1g PE=1 SV=3** | 373235.1 | 577674.7 | 0.65 |
| O09131 | **Glutathione S-transferase omega-1 OS=Mus musculus GN=Gsto1 PE=2 SV=2** | 622771.3 | 959762.0 | 0.65 |
| P61979 | **Heterogeneous nuclear ribonucleoprotein K OS=Mus musculus GN=Hnrnpk PE=1 SV=1** | 699525.7 | 1071123.5 | 0.65 |
| Q00896 | **Alpha-1-antitrypsin 1-3 OS=Mus musculus GN=Serpina1c PE=1 SV=2** | 132002.9 | 201752.0 | 0.65 |
| P19157 | **Glutathione S-transferase P 1 OS=Mus musculus GN=Gstp1 PE=1 SV=2** | 202015.7 | 308313.9 | 0.66 |
| P60229 | **Eukaryotic translation initiation factor 3 subunit E OS=Mus musculus GN=Eif3e PE=1 SV=1** | 4712.8 | 7183.8 | 0.66 |
| P84089 | **Enhancer of rudimentary homolog OS=Mus musculus GN=Erh PE=1 SV=1** | 86141.9 | 130924.1 | 0.66 |
| P97461 | **40S ribosomal protein S5 OS=Mus musculus GN=Rps5 PE=1 SV=3** | 116694.2 | 176430.5 | 0.66 |
| P14733 | **Lamin-B1 OS=Mus musculus GN=Lmnb1 PE=1 SV=3** | 216898.8 | 327543.5 | 0.66 |
| P00493 | **Hypoxanthine-guanine phosphoribosyltransferase OS=Mus musculus GN=Hprt1 PE=1 SV=3** | 52693.9 | 79392.1 | 0.66 |
| P16045 | **Galectin-1 OS=Mus musculus GN=Lgals1 PE=1 SV=3** | 3018754.8 | 4536266.2 | 0.67 |
| P50396 | **Rab GDP dissociation inhibitor alpha OS=Mus musculus GN=Gdi1 PE=1 SV=3** | 12752.6 | 19012.3 | 0.67 |
| Q9QXK3 | **Coatomer subunit gamma-2 OS=Mus musculus GN=Copg2 PE=2 SV=1** | 36768.2 | 54700.6 | 0.67 |
| Q9JIK5 | **Nucleolar RNA helicase 2 OS=Mus musculus GN=Ddx21 PE=1 SV=3** | 18765.3 | 27830.1 | 0.67 |
| P62774 | **Myotrophin OS=Mus musculus GN=Mtpn PE=1 SV=2** | 215681.0 | 319775.2 | 0.67 |
| P10649 | **Glutathione S-transferase Mu 1 OS=Mus musculus GN=Gstm1 PE=1 SV=2** | 57290.3 | 84547.5 | 0.68 |
| Q60854 | **Serpin B6 OS=Mus musculus GN=Serpinb6 PE=1 SV=1** | 883387.1 | 1301357.9 | 0.68 |
| Q9R0Q7 | **Prostaglandin E synthase 3 OS=Mus musculus GN=Ptges3 PE=1 SV=1** | 457585.5 | 669752.2 | 0.68 |
| Q6A4J8 | **Ubiquitin carboxyl-terminal hydrolase 7 OS=Mus musculus GN=Usp7 PE=1 SV=1** | 7913.0 | 11568.1 | 0.68 |
| Q9CY58 | **Plasminogen activator inhibitor 1 RNA-binding protein OS=Mus musculus GN=Serbp1 PE=1 SV=2** | 624111.3 | 912125.4 | 0.68 |
| Q64737 | **Trifunctional purine biosynthetic protein adenosine-3 OS=Mus musculus GN=Gart PE=2 SV=3** | 48601.7 | 70953.5 | 0.68 |
| P61971 | **Nuclear transport factor 2 OS=Mus musculus GN=Nutf2 PE=2 SV=1** | 79703.9 | 116159.0 | 0.69 |
| Q9WUM3 | **Coronin-1B OS=Mus musculus GN=Coro1b PE=1 SV=1** | 37228.7 | 54169.0 | 0.69 |
| Q7TPR4 | **Alpha-actinin-1 OS=Mus musculus GN=Actn1 PE=1 SV=1** | 234113.5 | 340393.2 | 0.69 |
| Q8BHN3 | **Neutral alpha-glucosidase AB OS=Mus musculus GN=Ganab PE=1 SV=1** | 10615.2 | 15404.0 | 0.69 |
| Q9JJU8 | **SH3 domain-binding glutamic acid-rich-like protein OS=Mus musculus GN=Sh3bgrl PE=3 SV=1** | 51710.7 | 75009.8 | 0.69 |
| P17225 | **Polypyrimidine tract-binding protein 1 OS=Mus musculus GN=Ptbp1 PE=1 SV=2** | 73827.3 | 106781.0 | 0.69 |
| Q62422 | **Osteoclast-stimulating factor 1 OS=Mus musculus GN=Ostf1 PE=1 SV=2** | 12517.9 | 17964.7 | 0.70 |

| | | | | |
|---|---|---|---|---|
| Q61074 | **Protein phosphatase 1G OS=Mus musculus GN=Ppm1g PE=1 SV=3** | 3395.7 | 4849.7 | 0.70 |
| P68254 | **14-3-3 protein theta OS=Mus musculus GN=Ywhaq PE=1 SV=1** | 591196.4 | 823121.6 | 0.72 |
| P26041 | **Moesin OS=Mus musculus GN=Msn PE=1 SV=3** | 315652.8 | 439327.4 | 0.72 |
| P43275 | **Histone H1.1 OS=Mus musculus GN=Hist1h1a PE=1 SV=2** | 105459.5 | 146761.5 | 0.72 |
| Q9QYB1 | **Chloride intracellular channel protein 4 OS=Mus musculus GN=Clic4 PE=1 SV=3** | 36362.8 | 50563.2 | 0.72 |
| Q99KQ4 | **Nicotinamide phosphoribosyltransferase OS=Mus musculus GN=Nampt PE=1 SV=1** | 11708.1 | 16274.7 | 0.72 |
| Q91YN5 | **UDP-N-acetylhexosamine pyrophosphorylase OS=Mus musculus GN=Uap1 PE=1 SV=1** | 17248.5 | 23917.8 | 0.72 |
| Q99K48 | **Non-POU domain-containing octamer-binding protein OS=Mus musculus GN=Nono PE=1 SV=3** | 24136.8 | 33440.2 | 0.72 |
| Q99JY9 | **Actin-related protein 3 OS=Mus musculus GN=Actr3 PE=1 SV=3** | 62830.9 | 86894.4 | 0.72 |
| Q8BFY9 | **Transportin-1 OS=Mus musculus GN=Tnpo1 PE=1 SV=2** | 34344.5 | 47464.1 | 0.72 |
| P60766 | **Cell division control protein 42 homolog OS=Mus musculus GN=Cdc42 PE=1 SV=2** | 59666.7 | 82358.5 | 0.72 |
| Q9Z1R2 | **Large proline-rich protein BAG6 OS=Mus musculus GN=Bag6 PE=1 SV=1** | 26540.8 | 36512.2 | 0.73 |
| P62983 | **Ubiquitin-40S ribosomal protein S27a OS=Mus musculus GN=Rps27a PE=1 SV=2** | 647974.1 | 890572.1 | 0.73 |
| Q8R1F1 | **Niban-like protein 1 OS=Mus musculus GN=Fam129b PE=2 SV=2** | 31606.5 | 43409.3 | 0.73 |
| P83870 | **PHD finger-like domain-containing protein 5A OS=Mus musculus GN=Phf5a PE=1 SV=1** | 27501.4 | 37696.2 | 0.73 |
| Q9DBP5 | **UMP-CMP kinase OS=Mus musculus GN=Cmpk1 PE=1 SV=1** | 17609.4 | 24128.9 | 0.73 |
| P70698 | **CTP synthase 1 OS=Mus musculus GN=Ctps1 PE=1 SV=2** | 40899.5 | 55816.1 | 0.73 |
| P68433 | **Histone H3.1 OS=Mus musculus GN=Hist1h3a PE=1 SV=2** | 252870.1 | 344753.0 | 0.73 |
| Q64337 | **Sequestosome-1 OS=Mus musculus GN=Sqstm1 PE=1 SV=1** | 63121.5 | 86005.8 | 0.73 |
| Q8K298 | **Actin-binding protein anillin OS=Mus musculus GN=Anln PE=1 SV=2** | 4177.4 | 5686.1 | 0.73 |
| Q3U898 | **Myeloma-overexpressed gene 2 protein homolog OS=Mus musculus GN=Myeov2 PE=3 SV=1** | 32073.1 | 43541.6 | 0.74 |
| Q6IRU2 | **Tropomyosin alpha-4 chain OS=Mus musculus GN=Tpm4 PE=2 SV=3** | 329527.6 | 444526.4 | 0.74 |
| P58044 | **Isopentenyl-diphosphate Delta-isomerase 1 OS=Mus musculus GN=Idi1 PE=2 SV=1** | 61448.6 | 82869.1 | 0.74 |
| Q6P5F9 | **Exportin-1 OS=Mus musculus GN=Xpo1 PE=1 SV=1** | 85198.2 | 114892.1 | 0.74 |
| Q8CAY6 | **Acetyl-CoA acetyltransferase, cytosolic OS=Mus musculus GN=Acat2 PE=1 SV=2** | 24832.5 | 33394.5 | 0.74 |
| Q93092 | **Transaldolase OS=Mus musculus GN=Taldo1 PE=1 SV=2** | 967758.7 | 1298268.1 | 0.75 |
| P62137 | **Serine/threonine-protein phosphatase PP1-alpha catalytic subunit OS=Mus musculus GN=Ppp1ca PE=1 SV=1** | 96944.6 | 130030.7 | 0.75 |
| P24452 | **Macrophage-capping protein OS=Mus musculus GN=Capg PE=1 SV=2** | 204787.3 | 273972.7 | 0.75 |
| P35700 | **Peroxiredoxin-1 OS=Mus musculus GN=Prdx1 PE=1 SV=1** | 3902848.6 | 5209184.1 | 0.75 |
| Q3TGF2 | **Protein FAM107B OS=Mus musculus GN=Fam107b PE=1 SV=2** | 9774.1 | 13038.9 | 0.75 |
| O35887 | **Calumenin OS=Mus musculus GN=Calu PE=1 SV=1** | 59747.4 | 79216.3 | 0.75 |
| Q8CGC7 | **Bifunctional glutamate/proline--tRNA ligase OS=Mus musculus GN=Eprs PE=1 SV=4** | 11780.0 | 15577.3 | 0.76 |
| P62196 | **26S protease regulatory subunit 8 OS=Mus musculus GN=Psmc5 PE=1 SV=1** | 10956.9 | 14480.0 | 0.76 |

| | | | | |
|---|---|---|---|---|
| Q9Z130 | **Heterogeneous nuclear ribonucleoprotein D-like OS=Mus musculus GN=Hnrnpdl PE=1 SV=1** | 20999.8 | 27710.2 | 0.76 |
| Q99KP6 | **Pre-mRNA-processing factor 19 OS=Mus musculus GN=Prpf19 PE=1 SV=1** | 111800.7 | 146966.2 | 0.76 |
| Q60668 | **Heterogeneous nuclear ribonucleoprotein D0 OS=Mus musculus GN=Hnrnpd PE=1 SV=2** | 400175.2 | 525071.7 | 0.76 |
| Q9DBR1 | **5'-3' exoribonuclease 2 OS=Mus musculus GN=Xrn2 PE=1 SV=1** | 13762.8 | 17912.1 | 0.77 |
| Q3TW96 | **UDP-N-acetylhexosamine pyrophosphorylase-like protein 1 OS=Mus musculus GN=Uap1l1 PE=2 SV=1** | 33089.0 | 42847.4 | 0.77 |
| P35979 | **60S ribosomal protein L12 OS=Mus musculus GN=Rpl12 PE=1 SV=2** | 139248.0 | 180186.9 | 0.77 |
| Q8BTM8 | **Filamin-A OS=Mus musculus GN=Flna PE=1 SV=5** | 1048550.0 | 1354080.6 | 0.77 |
| P58252 | **Elongation factor 2 OS=Mus musculus GN=Eef2 PE=1 SV=2** | 2591545.5 | 3338506.5 | 0.78 |
| P99027 | **60S acidic ribosomal protein P2 OS=Mus musculus GN=Rplp2 PE=1 SV=3** | 511278.8 | 657751.1 | 0.78 |
| Q62261 | **Spectrin beta chain, non-erythrocytic 1 OS=Mus musculus GN=Sptbn1 PE=1 SV=2** | 57376.1 | 73258.4 | 0.78 |
| Q9CR16 | **Peptidyl-prolyl cis-trans isomerase D OS=Mus musculus GN=Ppid PE=1 SV=3** | 86499.7 | 110274.6 | 0.78 |
| O08663 | **Methionine aminopeptidase 2 OS=Mus musculus GN=Metap2 PE=1 SV=1** | 6873.7 | 8732.7 | 0.79 |
| Q9CQC6 | **Basic leucine zipper and W2 domain-containing protein 1 OS=Mus musculus GN=Bzw1 PE=1 SV=1** | 41531.6 | 52719.1 | 0.79 |
| Q61553 | **Fascin OS=Mus musculus GN=Fscn1 PE=1 SV=4** | 865856.5 | 1098563.4 | 0.79 |
| Q62093 | **Serine/arginine-rich splicing factor 2 OS=Mus musculus GN=Srsf2 PE=1 SV=4** | 17676.0 | 22235.7 | 0.79 |
| Q501J6 | **Probable ATP-dependent RNA helicase DDX17 OS=Mus musculus GN=Ddx17 PE=1 SV=1** | 7025.7 | 8826.9 | 0.80 |
| O70194 | **Eukaryotic translation initiation factor 3 subunit D OS=Mus musculus GN=Eif3d PE=1 SV=2** | 46595.2 | 58282.1 | 0.80 |
| P10107 | **Annexin A1 OS=Mus musculus GN=Anxa1 PE=1 SV=2** | 806750.4 | 1008856.1 | 0.80 |
| Q9DCD0 | **6-phosphogluconate dehydrogenase, decarboxylating OS=Mus musculus GN=Pgd PE=1 SV=3** | 2339037.4 | 2923874.5 | 0.80 |
| P13864 | **DNA (cytosine-5)-methyltransferase 1 OS=Mus musculus GN=Dnmt1 PE=1 SV=5** | 8195.6 | 10233.0 | 0.80 |
| Q8BJW6 | **Eukaryotic translation initiation factor 2A OS=Mus musculus GN=Eif2a PE=2 SV=2** | 33443.2 | 41598.6 | 0.80 |
| Q8VIJ6 | **Splicing factor, proline- and glutamine-rich OS=Mus musculus GN=Sfpq PE=1 SV=1** | 192687.0 | 239596.4 | 0.80 |
| P62320 | **Small nuclear ribonucleoprotein Sm D3 OS=Mus musculus GN=Snrpd3 PE=1 SV=1** | 115523.8 | 143536.9 | 0.80 |
| Q9EQU5 | **Protein SET OS=Mus musculus GN=Set PE=1 SV=1** | 1751229.3 | 2161310.5 | 0.81 |
| P60335 | **Poly(rC)-binding protein 1 OS=Mus musculus GN=Pcbp1 PE=1 SV=1** | 77175.0 | 95092.6 | 0.81 |
| Q9WV32 | **Actin-related protein 2/3 complex subunit 1B OS=Mus musculus GN=Arpc1b PE=2 SV=4** | 84097.2 | 103532.6 | 0.81 |
| O35286 | **Pre-mRNA-splicing factor ATP-dependent RNA helicase DHX15 OS=Mus musculus GN=Dhx15 PE=1 SV=2** | 55246.8 | 67751.9 | 0.82 |
| P62307 | **Small nuclear ribonucleoprotein F OS=Mus musculus GN=Snrpf PE=3 SV=1** | 22001.3 | 26943.8 | 0.82 |
| P43277 | **Histone H1.3 OS=Mus musculus GN=Hist1h1d PE=1 SV=2** | 22770.5 | 27793.1 | 0.82 |
| Q9JMA1 | **Ubiquitin carboxyl-terminal hydrolase 14 OS=Mus musculus GN=Usp14 PE=1 SV=3** | 58053.8 | 70693.2 | 0.82 |
| P68037 | **Ubiquitin-conjugating enzyme E2 L3 OS=Mus musculus GN=Ube2l3 PE=2 SV=1** | 152515.0 | 184744.1 | 0.83 |

| | | | | |
|---|---|---|---|---|
| Q9JII5 | **DAZ-associated protein 1 OS=Mus musculus GN=Dazap1 PE=2 SV=2** | 54246.2 | 65700.5 | 0.83 |
| Q99KK7 | **Dipeptidyl peptidase 3 OS=Mus musculus GN=Dpp3 PE=2 SV=2** | 276814.8 | 334660.9 | 0.83 |
| P68040 | **Guanine nucleotide-binding protein subunit beta-2-like 1 OS=Mus musculus GN=Gnb2l1 PE=1 SV=3** | 2080791.2 | 2510883.8 | 0.83 |
| Q920B9 | **FACT complex subunit SPT16 OS=Mus musculus GN=Supt16h PE=1 SV=2** | 32893.1 | 39606.3 | 0.83 |
| P40124 | **Adenylyl cyclase-associated protein 1 OS=Mus musculus GN=Cap1 PE=1 SV=4** | 216290.1 | 260248.1 | 0.83 |
| P62852 | **40S ribosomal protein S25 OS=Mus musculus GN=Rps25 PE=1 SV=1** | 38968.0 | 46733.0 | 0.83 |
| Q80XU3 | **Nuclear ubiquitous casein and cyclin-dependent kinase substrate 1 OS=Mus musculus GN=Nucks1 PE=1 SV=1** | 42539.7 | 50908.5 | 0.84 |
| P97807 | **Fumarate hydratase, mitochondrial OS=Mus musculus GN=Fh PE=1 SV=3** | 33998.8 | 40665.0 | 0.84 |
| P28656 | **Nucleosome assembly protein 1-like 1 OS=Mus musculus GN=Nap1l1 PE=1 SV=2** | 568331.2 | 678236.9 | 0.84 |
| Q6P4T2 | **U5 small nuclear ribonucleoprotein 200 kDa helicase OS=Mus musculus GN=Snrnp200 PE=1 SV=1** | 4492.1 | 5344.8 | 0.84 |
| Q9QVP9 | **Protein-tyrosine kinase 2-beta OS=Mus musculus GN=Ptk2b PE=1 SV=2** | 25344.2 | 30013.3 | 0.84 |
| P40142 | **Transketolase OS=Mus musculus GN=Tkt PE=1 SV=1** | 3080374.1 | 3642222.6 | 0.85 |
| Q9CQL1 | **Protein mago nashi homolog 2 OS=Mus musculus GN=Magohb PE=2 SV=1** | 38408.9 | 45403.4 | 0.85 |
| P63101 | **14-3-3 protein zeta/delta OS=Mus musculus GN=Ywhaz PE=1 SV=1** | 4994451.2 | 5901385.0 | 0.85 |
| Q9Z0J0 | **Epididymal secretory protein E1 OS=Mus musculus GN=Npc2 PE=1 SV=1** | 27761.5 | 32781.6 | 0.85 |
| P35951 | **Low-density lipoprotein receptor OS=Mus musculus GN=Ldlr PE=1 SV=2** | 347175.7 | 409346.2 | 0.85 |
| P59999 | **Actin-related protein 2/3 complex subunit 4 OS=Mus musculus GN=Arpc4 PE=1 SV=3** | 33027.5 | 38849.7 | 0.85 |
| Q62446 | **Peptidyl-prolyl cis-trans isomerase FKBP3 OS=Mus musculus GN=Fkbp3 PE=1 SV=2** | 102465.4 | 120323.6 | 0.85 |
| Q9WUM4 | **Coronin-1C OS=Mus musculus GN=Coro1c PE=1 SV=2** | 21505.9 | 25019.1 | 0.86 |
| P62843 | **40S ribosomal protein S15 OS=Mus musculus GN=Rps15 PE=2 SV=2** | 73555.2 | 85532.4 | 0.86 |
| Q99LS3 | **Phosphoserine phosphatase OS=Mus musculus GN=Psph PE=2 SV=1** | 7574.6 | 8753.7 | 0.87 |
| Q91VM5 | **RNA binding motif protein, X-linked-like-1 OS=Mus musculus GN=Rbmxl1 PE=2 SV=1** | 145283.0 | 167890.0 | 0.87 |
| Q3UGC7 | **Eukaryotic translation initiation factor 3 subunit J-A OS=Mus musculus GN=Eif3j1 PE=2 SV=1** | 15307.9 | 17677.8 | 0.87 |
| P30416 | **Peptidyl-prolyl cis-trans isomerase FKBP4 OS=Mus musculus GN=Fkbp4 PE=1 SV=5** | 347800.4 | 401601.6 | 0.87 |
| Q8JZK9 | **Hydroxymethylglutaryl-CoA synthase, cytoplasmic OS=Mus musculus GN=Hmgcs1 PE=1 SV=1** | 63545.1 | 73038.3 | 0.87 |
| P62270 | **40S ribosomal protein S18 OS=Mus musculus GN=Rps18 PE=1 SV=3** | 190789.1 | 219244.7 | 0.87 |
| Q61990 | **Poly(rC)-binding protein 2 OS=Mus musculus GN=Pcbp2 PE=1 SV=1** | 137668.2 | 158169.5 | 0.87 |
| O08997 | **Copper transport protein ATOX1 OS=Mus musculus GN=Atox1 PE=1 SV=1** | 347990.9 | 399442.9 | 0.87 |
| Q9JJV2 | **Profilin-2 OS=Mus musculus GN=Pfn2 PE=1 SV=3** | 23094.2 | 26420.3 | 0.87 |
| P41105 | **60S ribosomal protein L28 OS=Mus musculus GN=Rpl28 PE=1 SV=2** | 25438.3 | 29052.6 | 0.88 |

| | | | | |
|---|---|---|---|---|
| P62858 | **40S ribosomal protein S28 OS=Mus musculus GN=Rps28 PE=3 SV=1** | 676852.2 | 772841.5 | 0.88 |
| Q99MD9 | **Nuclear autoantigenic sperm protein OS=Mus musculus GN=Nasp PE=1 SV=2** | 1174693.0 | 1340861.8 | 0.88 |
| P70296 | **Phosphatidylethanolamine-binding protein 1 OS=Mus musculus GN=Pebp1 PE=1 SV=3** | 1952975.2 | 2228034.6 | 0.88 |
| P57780 | **Alpha-actinin-4 OS=Mus musculus GN=Actn4 PE=1 SV=1** | 869161.9 | 991069.3 | 0.88 |
| P17742 | **Peptidyl-prolyl cis-trans isomerase A OS=Mus musculus GN=Ppia PE=1 SV=2** | 10580392.0 | 12016047.6 | 0.88 |
| P34884 | **Macrophage migration inhibitory factor OS=Mus musculus GN=Mif PE=1 SV=2** | 2078793.0 | 2360144.7 | 0.88 |
| P62715 | **Serine/threonine-protein phosphatase 2A catalytic subunit beta isoform OS=Mus musculus GN=Ppp2cb PE=1 SV=1** | 139290.8 | 157915.9 | 0.88 |
| P29341 | **Polyadenylate-binding protein 1 OS=Mus musculus GN=Pabpc1 PE=1 SV=2** | 609712.9 | 689844.8 | 0.88 |
| Q9CQ65 | **S-methyl-5'-thioadenosine phosphorylase OS=Mus musculus GN=Mtap PE=1 SV=1** | 1133508.4 | 1279023.6 | 0.89 |
| Q78ZA7 | **Nucleosome assembly protein 1-like 4 OS=Mus musculus GN=Nap1l4 PE=1 SV=1** | 36307.8 | 40891.0 | 0.89 |
| P11499 | **Heat shock protein HSP 90-beta OS=Mus musculus GN=Hsp90ab1 PE=1 SV=3** | 7221912.5 | 8129267.3 | 0.89 |
| P61205 | **ADP-ribosylation factor 3 OS=Mus musculus GN=Arf3 PE=2 SV=2** | 17477.8 | 19560.8 | 0.89 |
| Q8BFZ3 | **Beta-actin-like protein 2 OS=Mus musculus GN=Actbl2 PE=1 SV=1** | 206818.8 | 231445.6 | 0.89 |
| Q9CZM2 | **60S ribosomal protein L15 OS=Mus musculus GN=Rpl15 PE=2 SV=4** | 204530.0 | 228791.8 | 0.89 |
| P16110 | **Galectin-3 OS=Mus musculus GN=Lgals3 PE=1 SV=3** | 587825.3 | 656738.9 | 0.90 |
| Q6ZWN5 | **40S ribosomal protein S9 OS=Mus musculus GN=Rps9 PE=1 SV=3** | 363942.3 | 406371.0 | 0.90 |
| P63017 | **Heat shock cognate 71 kDa protein OS=Mus musculus GN=Hspa8 PE=1 SV=1** | 25894799.3 | 28892575.8 | 0.90 |
| P51859 | **Hepatoma-derived growth factor OS=Mus musculus GN=Hdgf PE=1 SV=2** | 632212.1 | 704255.3 | 0.90 |
| P97379 | **Ras GTPase-activating protein-binding protein 2 OS=Mus musculus GN=G3bp2 PE=1 SV=2** | 23339.6 | 25892.0 | 0.90 |
| P80317 | **T-complex protein 1 subunit zeta OS=Mus musculus GN=Cct6a PE=1 SV=3** | 270786.5 | 300306.1 | 0.90 |
| P10711 | **Transcription elongation factor A protein 1 OS=Mus musculus GN=Tcea1 PE=1 SV=2** | 27003.2 | 29946.8 | 0.90 |
| P62317 | **Small nuclear ribonucleoprotein Sm D2 OS=Mus musculus GN=Snrpd2 PE=3 SV=1** | 23867.7 | 26403.5 | 0.90 |
| Q921F2 | **TAR DNA-binding protein 43 OS=Mus musculus GN=Tardbp PE=1 SV=1** | 658925.7 | 728333.2 | 0.90 |
| O35423 | **Serine--pyruvate aminotransferase, mitochondrial OS=Mus musculus GN=Agxt PE=1 SV=3** | 30452.6 | 33598.1 | 0.91 |
| P70372 | **ELAV-like protein 1 OS=Mus musculus GN=Elavl1 PE=1 SV=2** | 33730.5 | 37191.7 | 0.91 |
| Q9Z1Z2 | **Serine-threonine kinase receptor-associated protein OS=Mus musculus GN=Strap PE=1 SV=2** | 45464.6 | 50057.2 | 0.91 |
| Q9CVB6 | **Actin-related protein 2/3 complex subunit 2 OS=Mus musculus GN=Arpc2 PE=1 SV=3** | 25266.1 | 27744.4 | 0.91 |
| P50543 | **Protein S100-A11 OS=Mus musculus GN=S100a11 PE=1 SV=1** | 1483014.4 | 1626383.0 | 0.91 |
| P62908 | **40S ribosomal protein S3 OS=Mus musculus GN=Rps3 PE=1 SV=1** | 342201.7 | 375138.0 | 0.91 |

| | | | | |
|---|---|---|---|---|
| Q9QZD9 | **Eukaryotic translation initiation factor 3 subunit I OS=Mus musculus GN=Eif3i PE=1 SV=1** | 19222.4 | 21048.8 | 0.91 |
| Q61206 | **Platelet-activating factor acetylhydrolase IB subunit beta OS=Mus musculus GN=Pafah1b2 PE=1 SV=2** | 26362.4 | 28867.3 | 0.91 |
| Q61081 | **Hsp90 co-chaperone Cdc37 OS=Mus musculus GN=Cdc37 PE=2 SV=1** | 126035.0 | 137955.3 | 0.91 |
| Q9QYJ0 | **DnaJ homolog subfamily A member 2 OS=Mus musculus GN=Dnaja2 PE=1 SV=1** | 5099.7 | 5573.2 | 0.92 |
| Q61753 | **D-3-phosphoglycerate dehydrogenase OS=Mus musculus GN=Phgdh PE=1 SV=3** | 88638.2 | 96812.2 | 0.92 |
| Q9Z2N8 | **Actin-like protein 6A OS=Mus musculus GN=Actl6a PE=1 SV=2** | 27478.5 | 30001.8 | 0.92 |
| P80313 | **T-complex protein 1 subunit eta OS=Mus musculus GN=Cct7 PE=1 SV=1** | 726807.6 | 792956.4 | 0.92 |
| P26040 | **Ezrin OS=Mus musculus GN=Ezr PE=1 SV=3** | 133852.8 | 145782.9 | 0.92 |
| P06801 | **NADP-dependent malic enzyme OS=Mus musculus GN=Me1 PE=1 SV=2** | 108756.9 | 118321.6 | 0.92 |
| Q8BP47 | **Asparagine--tRNA ligase, cytoplasmic OS=Mus musculus GN=Nars PE=1 SV=2** | 111038.0 | 120763.5 | 0.92 |
| P14131 | **40S ribosomal protein S16 OS=Mus musculus GN=Rps16 PE=2 SV=4** | 155488.0 | 168879.6 | 0.92 |
| P80318 | **T-complex protein 1 subunit gamma OS=Mus musculus GN=Cct3 PE=1 SV=1** | 835951.3 | 906778.7 | 0.92 |
| Q91W90 | **Thioredoxin domain-containing protein 5 OS=Mus musculus GN=Txndc5 PE=1 SV=2** | 8652.0 | 9366.7 | 0.92 |
| Q9CT10 | **Ran-binding protein 3 OS=Mus musculus GN=Ranbp3 PE=1 SV=2** | 5695.6 | 6145.9 | 0.93 |
| Q9JIF0 | **Protein arginine N-methyltransferase 1 OS=Mus musculus GN=Prmt1 PE=1 SV=1** | 143784.0 | 154656.4 | 0.93 |
| P62702 | **40S ribosomal protein S4, X isoform OS=Mus musculus GN=Rps4x PE=2 SV=2** | 262962.5 | 281901.8 | 0.93 |
| P24547 | **Inosine-5'-monophosphate dehydrogenase 2 OS=Mus musculus GN=Impdh2 PE=1 SV=2** | 12405.0 | 13267.1 | 0.94 |
| P63242 | **Eukaryotic translation initiation factor 5A-1 OS=Mus musculus GN=Eif5a PE=1 SV=2** | 1512429.4 | 1615296.0 | 0.94 |
| Q9WVA3 | **Mitotic checkpoint protein BUB3 OS=Mus musculus GN=Bub3 PE=2 SV=2** | 12643.9 | 13502.0 | 0.94 |
| Q7TMK9 | **Heterogeneous nuclear ribonucleoprotein Q OS=Mus musculus GN=Syncrip PE=1 SV=2** | 241468.2 | 257552.8 | 0.94 |
| P68510 | **14-3-3 protein eta OS=Mus musculus GN=Ywhah PE=1 SV=2** | 486778.5 | 519145.3 | 0.94 |
| P62264 | **40S ribosomal protein S14 OS=Mus musculus GN=Rps14 PE=2 SV=3** | 24693.9 | 26321.7 | 0.94 |
| P26883 | **Peptidyl-prolyl cis-trans isomerase FKBP1A OS=Mus musculus GN=Fkbp1a PE=1 SV=2** | 626020.4 | 666695.8 | 0.94 |
| Q9CQM5 | **Thioredoxin domain-containing protein 17 OS=Mus musculus GN=Txndc17 PE=1 SV=1** | 215816.5 | 229559.3 | 0.94 |
| Q99K85 | **Phosphoserine aminotransferase OS=Mus musculus GN=Psat1 PE=1 SV=1** | 377258.9 | 401238.9 | 0.94 |
| P47879 | **Insulin-like growth factor-binding protein 4 OS=Mus musculus GN=Igfbp4 PE=2 SV=2** | 10140.6 | 10783.3 | 0.94 |
| Q00612 | **Glucose-6-phosphate 1-dehydrogenase X OS=Mus musculus GN=G6pdx PE=1 SV=3** | 28757.2 | 30576.3 | 0.94 |
| Q9D7S9 | **Charged multivesicular body protein 5 OS=Mus musculus GN=Chmp5 PE=2 SV=1** | 44074.0 | 46774.6 | 0.94 |
| Q8BWZ3 | **N-alpha-acetyltransferase 25, NatB auxiliary subunit OS=Mus musculus GN=Naa25 PE=2 SV=1** | 8980.6 | 9528.8 | 0.94 |

| | | | | |
|---|---|---|---|---|
| P13597 | **Intercellular adhesion molecule 1 OS=Mus musculus GN=Icam1 PE=1 SV=1** | 5878.0 | 6227.1 | 0.94 |
| Q6ZWU9 | **40S ribosomal protein S27 OS=Mus musculus GN=Rps27 PE=1 SV=3** | 121394.5 | 128574.1 | 0.94 |
| Q9Z2X1 | **Heterogeneous nuclear ribonucleoprotein F OS=Mus musculus GN=Hnrnpf PE=1 SV=3** | 301628.8 | 319262.6 | 0.94 |
| Q9WTX6 | **Cullin-1 OS=Mus musculus GN=Cul1 PE=1 SV=1** | 6511.0 | 6888.9 | 0.95 |
| P02468 | **Laminin subunit gamma-1 OS=Mus musculus GN=Lamc1 PE=1 SV=2** | 61347.0 | 64825.1 | 0.95 |
| P26039 | **Talin-1 OS=Mus musculus GN=Tln1 PE=1 SV=2** | 241487.6 | 254791.4 | 0.95 |
| Q61029 | **Lamina-associated polypeptide 2, isoforms beta/delta/epsilon/gamma OS=Mus musculus GN=Tmpo PE=1 SV=4** | 87359.5 | 91908.3 | 0.95 |
| Q60973 | **Histone-binding protein RBBP7 OS=Mus musculus GN=Rbbp7 PE=1 SV=1** | 353724.3 | 370130.3 | 0.96 |
| Q6PGH2 | **Hematological and neurological expressed 1-like protein OS=Mus musculus GN=Hn1l PE=1 SV=1** | 24461.4 | 25556.2 | 0.96 |
| Q8K310 | **Matrin-3 OS=Mus musculus GN=Matr3 PE=1 SV=1** | 175703.1 | 182771.0 | 0.96 |
| P67984 | **60S ribosomal protein L22 OS=Mus musculus GN=Rpl22 PE=1 SV=2** | 32866.0 | 34168.3 | 0.96 |
| Q9D8E6 | **60S ribosomal protein L4 OS=Mus musculus GN=Rpl4 PE=1 SV=3** | 395059.4 | 410548.5 | 0.96 |
| P14873 | **Microtubule-associated protein 1B OS=Mus musculus GN=Map1b PE=1 SV=2** | 241528.0 | 250700.9 | 0.96 |
| Q9CQR2 | **40S ribosomal protein S21 OS=Mus musculus GN=Rps21 PE=3 SV=1** | 435060.3 | 450795.0 | 0.97 |
| Q9JKB3 | **Y-box-binding protein 3 OS=Mus musculus GN=Ybx3 PE=1 SV=2** | 28681.8 | 29695.4 | 0.97 |
| P26369 | **Splicing factor U2AF 65 kDa subunit OS=Mus musculus GN=U2af2 PE=1 SV=3** | 260603.8 | 268544.3 | 0.97 |
| Q91V12 | **Cytosolic acyl coenzyme A thioester hydrolase OS=Mus musculus GN=Acot7 PE=1 SV=2** | 19416.2 | 19995.6 | 0.97 |
| Q8CDN6 | **Thioredoxin-like protein 1 OS=Mus musculus GN=Txnl1 PE=2 SV=3** | 214146.9 | 220266.5 | 0.97 |
| P07214 | **SPARC OS=Mus musculus GN=Sparc PE=1 SV=1** | 957828.3 | 985146.7 | 0.97 |
| Q9CPY7 | **Cytosol aminopeptidase OS=Mus musculus GN=Lap3 PE=1 SV=3** | 83678.3 | 85887.9 | 0.97 |
| P61161 | **Actin-related protein 2 OS=Mus musculus GN=Actr2 PE=1 SV=1** | 36202.6 | 37087.8 | 0.98 |
| Q9CR41 | **Huntingtin-interacting protein K OS=Mus musculus GN=Hypk PE=2 SV=2** | 24014.9 | 24589.3 | 0.98 |
| P60710 | **Actin, cytoplasmic 1 OS=Mus musculus GN=Actb PE=1 SV=1** | 2352435.3 | 2405039.8 | 0.98 |
| P70349 | **Histidine triad nucleotide-binding protein 1 OS=Mus musculus GN=Hint1 PE=1 SV=3** | 130002.2 | 132872.4 | 0.98 |
| O55131 | **Septin-7 OS=Mus musculus GN=Sept7 PE=1 SV=1** | 26513.4 | 27087.8 | 0.98 |
| O35685 | **Nuclear migration protein nudC OS=Mus musculus GN=Nudc PE=1 SV=1** | 22249.5 | 22708.8 | 0.98 |
| Q8VEK3 | **Heterogeneous nuclear ribonucleoprotein U OS=Mus musculus GN=Hnrnpu PE=1 SV=1** | 455882.7 | 464163.2 | 0.98 |
| Q8BIQ5 | **Cleavage stimulation factor subunit 2 OS=Mus musculus GN=Cstf2 PE=1 SV=2** | 6632.9 | 6747.3 | 0.98 |
| P54822 | **Adenylosuccinate lyase OS=Mus musculus GN=Adsl PE=2 SV=2** | 14459.8 | 14700.9 | 0.98 |
| Q9JLV1 | **BAG family molecular chaperone regulator 3 OS=Mus musculus GN=Bag3 PE=1 SV=2** | 94980.0 | 96512.4 | 0.98 |

| | | | | |
|---|---|---|---|---|
| Q9CZ04 | **COP9 signalosome complex subunit 7a OS=Mus musculus GN=Cops7a PE=1 SV=2** | 4650.4 | 4709.0 | 0.99 |
| P43274 | **Histone H1.4 OS=Mus musculus GN=Hist1h1e PE=1 SV=2** | 165444.2 | 166922.9 | 0.99 |
| P07901 | **Heat shock protein HSP 90-alpha OS=Mus musculus GN=Hsp90aa1 PE=1 SV=4** | 5875152.3 | 5914369.1 | 0.99 |
| Q6ZWY9 | **Histone H2B type 1-C/E/G OS=Mus musculus GN=Hist1h2bc PE=1 SV=3** | 1219133.9 | 1226117.0 | 0.99 |
| Q9QZ73 | **DCN1-like protein 1 OS=Mus musculus GN=Dcun1d1 PE=2 SV=1** | 10974.4 | 11001.3 | 1.00 |
| P42669 | **Transcriptional activator protein Pur-alpha OS=Mus musculus GN=Pura PE=1 SV=1** | 41569.5 | 41668.4 | 1.00 |
| Q68FD5 | **Clathrin heavy chain 1 OS=Mus musculus GN=Cltc PE=1 SV=3** | 289504.1 | 289924.5 | 1.00 |
| F6ZDS4 | **Nucleoprotein TPR OS=Mus musculus GN=Tpr PE=1 SV=1** | 5207.5 | 5195.2 | 1.00 |
| P17918 | **Proliferating cell nuclear antigen OS=Mus musculus GN=Pcna PE=1 SV=2** | 754485.0 | 751250.8 | 1.00 |
| P25444 | **40S ribosomal protein S2 OS=Mus musculus GN=Rps2 PE=2 SV=3** | 248734.8 | 247346.5 | 1.01 |
| Q60605 | **Myosin light polypeptide 6 OS=Mus musculus GN=Myl6 PE=1 SV=3** | 225022.5 | 223764.3 | 1.01 |
| Q8BMJ3 | **Eukaryotic translation initiation factor 1A, X-chromosomal OS=Mus musculus GN=Eif1ax PE=2 SV=3** | 242034.9 | 240356.1 | 1.01 |
| Q76MZ3 | **Serine/threonine-protein phosphatase 2A 65 kDa regulatory subunit A alpha isoform OS=Mus musculus GN=Ppp2r1a PE=1 SV=3** | 307021.7 | 304733.6 | 1.01 |
| P23198 | **Chromobox protein homolog 3 OS=Mus musculus GN=Cbx3 PE=1 SV=2** | 218485.9 | 216811.0 | 1.01 |
| Q9DAR7 | **m7GpppX diphosphatase OS=Mus musculus GN=Dcps PE=1 SV=1** | 135015.0 | 133851.9 | 1.01 |
| Q60817 | **Nascent polypeptide-associated complex subunit alpha OS=Mus musculus GN=Naca PE=1 SV=1** | 426887.9 | 422907.0 | 1.01 |
| P61982 | **14-3-3 protein gamma OS=Mus musculus GN=Ywhag PE=1 SV=2** | 625512.4 | 618494.4 | 1.01 |
| P52912 | **Nucleolysin TIA-1 OS=Mus musculus GN=Tia1 PE=1 SV=1** | 18664.8 | 18377.7 | 1.02 |
| Q7TPV4 | **Myb-binding protein 1A OS=Mus musculus GN=Mybbp1a PE=1 SV=2** | 56291.4 | 54991.3 | 1.02 |
| Q9JMH6 | **Thioredoxin reductase 1, cytoplasmic OS=Mus musculus GN=Txnrd1 PE=1 SV=3** | 65682.6 | 64135.6 | 1.02 |
| P62717 | **60S ribosomal protein L18a OS=Mus musculus GN=Rpl18a PE=1 SV=1** | 61585.3 | 60081.8 | 1.03 |
| O09172 | **Glutamate--cysteine ligase regulatory subunit OS=Mus musculus GN=Gclm PE=2 SV=1** | 169030.1 | 164474.7 | 1.03 |
| Q9D358 | **Low molecular weight phosphotyrosine protein phosphatase OS=Mus musculus GN=Acp1 PE=1 SV=3** | 186605.5 | 181376.2 | 1.03 |
| Q6ZWX6 | **Eukaryotic translation initiation factor 2 subunit 1 OS=Mus musculus GN=Eif2s1 PE=1 SV=3** | 90904.9 | 88308.0 | 1.03 |
| Q9DCG9 | **Multifunctional methyltransferase subunit TRM112-like protein OS=Mus musculus GN=Trmt112 PE=2 SV=1** | 9647.2 | 9370.1 | 1.03 |
| P84099 | **60S ribosomal protein L19 OS=Mus musculus GN=Rpl19 PE=1 SV=1** | 150832.9 | 146384.0 | 1.03 |
| Q9D0I9 | **Arginine--tRNA ligase, cytoplasmic OS=Mus musculus GN=Rars PE=2 SV=2** | 79331.7 | 76971.2 | 1.03 |
| Q61598 | **Rab GDP dissociation inhibitor beta OS=Mus musculus GN=Gdi2 PE=1 SV=1** | 694702.3 | 673746.5 | 1.03 |
| Q9Z0N1 | **Eukaryotic translation initiation factor 2 subunit 3, X-linked OS=Mus musculus GN=Eif2s3x PE=1 SV=2** | 138024.6 | 133575.5 | 1.03 |

| | | | | |
|---|---|---|---|---|
| Q8BGD9 | **Eukaryotic translation initiation factor 4B OS=Mus musculus GN=Eif4b PE=1 SV=1** | 142983.2 | 138148.5 | 1.03 |
| P63158 | **High mobility group protein B1 OS=Mus musculus GN=Hmgb1 PE=1 SV=2** | 1494684.6 | 1434592.9 | 1.04 |
| Q60865 | **Caprin-1 OS=Mus musculus GN=Caprin1 PE=1 SV=2** | 504536.8 | 483803.8 | 1.04 |
| Q9JKF1 | **Ras GTPase-activating-like protein IQGAP1 OS=Mus musculus GN=Iqgap1 PE=1 SV=2** | 9630.9 | 9234.2 | 1.04 |
| Q9CWJ9 | **Bifunctional purine biosynthesis protein PURH OS=Mus musculus GN=Atic PE=1 SV=2** | 4652.4 | 4452.3 | 1.04 |
| Q8C1A5 | **Thimet oligopeptidase OS=Mus musculus GN=Thop1 PE=1 SV=1** | 137831.7 | 131790.3 | 1.05 |
| Q9CZ30 | **Obg-like ATPase 1 OS=Mus musculus GN=Ola1 PE=1 SV=1** | 124528.9 | 118930.5 | 1.05 |
| O08709 | **Peroxiredoxin-6 OS=Mus musculus GN=Prdx6 PE=1 SV=3** | 4561406.7 | 4335650.8 | 1.05 |
| P32067 | **Lupus La protein homolog OS=Mus musculus GN=Ssb PE=1 SV=1** | 195881.8 | 186044.8 | 1.05 |
| Q64695 | **Endothelial protein C receptor OS=Mus musculus GN=Procr PE=2 SV=3** | 255488.3 | 242433.6 | 1.05 |
| P46471 | **26S protease regulatory subunit 7 OS=Mus musculus GN=Psmc2 PE=1 SV=5** | 20647.7 | 19474.3 | 1.06 |
| P09411 | **Phosphoglycerate kinase 1 OS=Mus musculus GN=Pgk1 PE=1 SV=4** | 8692070.5 | 8197270.0 | 1.06 |
| P23116 | **Eukaryotic translation initiation factor 3 subunit A OS=Mus musculus GN=Eif3a PE=1 SV=5** | 16478.5 | 15527.5 | 1.06 |
| P27773 | **Protein disulfide-isomerase A3 OS=Mus musculus GN=Pdia3 PE=1 SV=2** | 811571.2 | 763441.1 | 1.06 |
| P63165 | **Contaminant (Small ubiquitin-related modifier 1)** | 26299.9 | 24693.5 | 1.07 |
| P53996 | **Cellular nucleic acid-binding protein OS=Mus musculus GN=Cnbp PE=1 SV=2** | 249944.8 | 234410.4 | 1.07 |
| Q61035 | **Histidine--tRNA ligase, cytoplasmic OS=Mus musculus GN=Hars PE=2 SV=2** | 10119.3 | 9482.2 | 1.07 |
| P62827 | **GTP-binding nuclear protein Ran OS=Mus musculus GN=Ran PE=1 SV=3** | 1304451.9 | 1221446.7 | 1.07 |
| P68372 | **Tubulin beta-4B chain OS=Mus musculus GN=Tubb4b PE=1 SV=1** | 192109.7 | 179855.1 | 1.07 |
| Q80X90 | **Filamin-B OS=Mus musculus GN=Flnb PE=1 SV=3** | 1271463.7 | 1187282.7 | 1.07 |
| P62897 | **Cytochrome c, somatic OS=Mus musculus GN=Cycs PE=1 SV=2** | 709651.3 | 661706.9 | 1.07 |
| P97822 | **Acidic leucine-rich nuclear phosphoprotein 32 family member E OS=Mus musculus GN=Anp32e PE=1 SV=2** | 373521.3 | 347366.9 | 1.08 |
| Q91WJ8 | **Far upstream element-binding protein 1 OS=Mus musculus GN=Fubp1 PE=1 SV=1** | 60631.6 | 56337.3 | 1.08 |
| P60867 | **40S ribosomal protein S20 OS=Mus musculus GN=Rps20 PE=1 SV=1** | 114073.2 | 105947.7 | 1.08 |
| Q9R1P0 | **Proteasome subunit alpha type-4 OS=Mus musculus GN=Psma4 PE=1 SV=1** | 352487.0 | 327214.7 | 1.08 |
| Q6NZJ6 | **Eukaryotic translation initiation factor 4 gamma 1 OS=Mus musculus GN=Eif4g1 PE=1 SV=1** | 81636.3 | 75629.6 | 1.08 |
| P99029 | **Peroxiredoxin-5, mitochondrial OS=Mus musculus GN=Prdx5 PE=1 SV=2** | 4935.1 | 4568.3 | 1.08 |
| P49312 | **Heterogeneous nuclear ribonucleoprotein A1 OS=Mus musculus GN=Hnrnpa1 PE=1 SV=2** | 2817766.2 | 2607190.5 | 1.08 |
| Q9R0P3 | **S-formylglutathione hydrolase OS=Mus musculus GN=Esd PE=1 SV=1** | 1148944.1 | 1063068.6 | 1.08 |
| Q99020 | **Heterogeneous nuclear ribonucleoprotein A/B OS=Mus musculus GN=Hnrnpab PE=1 SV=1** | 1424495.6 | 1314070.9 | 1.08 |

| | | | | |
|---|---|---|---|---|
| P19253 | **60S ribosomal protein L13a OS=Mus musculus GN=Rpl13a PE=1 SV=4** | 141249.8 | 130250.9 | 1.08 |
| Q8R1B4 | **Eukaryotic translation initiation factor 3 subunit C OS=Mus musculus GN=Eif3c PE=1 SV=1** | 38275.0 | 35167.8 | 1.09 |
| P62242 | **40S ribosomal protein S8 OS=Mus musculus GN=Rps8 PE=1 SV=2** | 1463238.6 | 1343734.4 | 1.09 |
| Q4KML4 | **Costars family protein ABRACL OS=Mus musculus GN=Abracl PE=3 SV=1** | 82963.5 | 76092.3 | 1.09 |
| P56959 | **RNA-binding protein FUS OS=Mus musculus GN=Fus PE=2 SV=1** | 516814.8 | 472372.6 | 1.09 |
| P62751 | **60S ribosomal protein L23a OS=Mus musculus GN=Rpl23a PE=1 SV=1** | 122611.8 | 112036.4 | 1.09 |
| Q9WVA2 | **Mitochondrial import inner membrane translocase subunit Tim8 A OS=Mus musculus GN=Timm8a1 PE=1 SV=1** | 50184.5 | 45851.8 | 1.09 |
| P52480 | **Pyruvate kinase PKM OS=Mus musculus GN=Pkm PE=1 SV=4** | 14080086.6 | 12856243.7 | 1.10 |
| P63168 | **Dynein light chain 1, cytoplasmic OS=Mus musculus GN=Dynll1 PE=1 SV=1** | 82200.9 | 74969.4 | 1.10 |
| P62855 | **40S ribosomal protein S26 OS=Mus musculus GN=Rps26 PE=3 SV=3** | 48903.6 | 44493.6 | 1.10 |
| Q01853 | **Transitional endoplasmic reticulum ATPase OS=Mus musculus GN=Vcp PE=1 SV=4** | 2299793.7 | 2089026.5 | 1.10 |
| P06745 | **Glucose-6-phosphate isomerase OS=Mus musculus GN=Gpi PE=1 SV=4** | 5531780.8 | 5017979.9 | 1.10 |
| Q8VDD5 | **Myosin-9 OS=Mus musculus GN=Myh9 PE=1 SV=4** | 145783.6 | 132195.6 | 1.10 |
| P24527 | **Leukotriene A-4 hydrolase OS=Mus musculus GN=Lta4h PE=1 SV=4** | 103365.3 | 93595.1 | 1.10 |
| P97352 | **Protein S100-A13 OS=Mus musculus GN=S100a13 PE=1 SV=1** | 75884.6 | 68643.2 | 1.11 |
| Q01320 | **DNA topoisomerase 2-alpha OS=Mus musculus GN=Top2a PE=1 SV=2** | 13822.1 | 12482.7 | 1.11 |
| P62281 | **40S ribosomal protein S11 OS=Mus musculus GN=Rps11 PE=1 SV=3** | 181856.8 | 163910.8 | 1.11 |
| P47964 | **60S ribosomal protein L36 OS=Mus musculus GN=Rpl36 PE=3 SV=2** | 70964.1 | 63887.6 | 1.11 |
| P97310 | **DNA replication licensing factor MCM2 OS=Mus musculus GN=Mcm2 PE=1 SV=3** | 4263.8 | 3824.1 | 1.11 |
| Q9Z2W0 | **Aspartyl aminopeptidase OS=Mus musculus GN=Dnpep PE=2 SV=2** | 11223.0 | 10062.3 | 1.12 |
| P48678 | **Prelamin-A/C OS=Mus musculus GN=Lmna PE=1 SV=2** | 2722596.6 | 2439687.7 | 1.12 |
| P14152 | **Malate dehydrogenase, cytoplasmic OS=Mus musculus GN=Mdh1 PE=1 SV=3** | 840074.3 | 751864.3 | 1.12 |
| Q02053 | **Ubiquitin-like modifier-activating enzyme 1 OS=Mus musculus GN=Uba1 PE=1 SV=1** | 275012.8 | 245860.5 | 1.12 |
| P62849 | **40S ribosomal protein S24 OS=Mus musculus GN=Rps24 PE=2 SV=1** | 19885.8 | 17749.4 | 1.12 |
| P47791 | **Glutathione reductase, mitochondrial OS=Mus musculus GN=Gsr PE=1 SV=3** | 324801.7 | 289864.8 | 1.12 |
| P08113 | **Endoplasmin OS=Mus musculus GN=Hsp90b1 PE=1 SV=2** | 49355.7 | 44038.2 | 1.12 |
| Q60692 | **Proteasome subunit beta type-6 OS=Mus musculus GN=Psmb6 PE=1 SV=3** | 1137009.7 | 1013062.6 | 1.12 |
| Q9DAK9 | **14 kDa phosphohistidine phosphatase OS=Mus musculus GN=Phpt1 PE=2 SV=1** | 14050.6 | 12513.1 | 1.12 |
| Q6ZQ38 | **Cullin-associated NEDD8-dissociated protein 1 OS=Mus musculus GN=Cand1 PE=2 SV=2** | 75271.6 | 67005.2 | 1.12 |
| O08585 | **Clathrin light chain A OS=Mus musculus GN=Clta PE=1 SV=2** | 109859.3 | 97743.7 | 1.12 |

| | | | | |
|---|---|---|---|---|
| Q62318 | **Transcription intermediary factor 1-beta OS=Mus musculus GN=Trim28 PE=1 SV=3** | 31563.2 | 28002.9 | 1.13 |
| P62806 | **Histone H4 OS=Mus musculus GN=Hist1h4a PE=1 SV=2** | 2404078.6 | 2130535.3 | 1.13 |
| Q8CHP8 | **Phosphoglycolate phosphatase OS=Mus musculus GN=Pgp PE=1 SV=1** | 81654.2 | 72025.2 | 1.13 |
| Q05144 | **Ras-related C3 botulinum toxin substrate 2 OS=Mus musculus GN=Rac2 PE=2 SV=1** | 25442.6 | 22427.5 | 1.13 |
| Q9JKB1 | **Ubiquitin carboxyl-terminal hydrolase isozyme L3 OS=Mus musculus GN=Uchl3 PE=1 SV=2** | 40090.7 | 35256.4 | 1.14 |
| Q9CWZ3 | **RNA-binding protein 8A OS=Mus musculus GN=Rbm8a PE=1 SV=3** | 44735.3 | 39337.3 | 1.14 |
| Q921M3 | **Splicing factor 3B subunit 3 OS=Mus musculus GN=Sf3b3 PE=2 SV=1** | 272514.0 | 239096.8 | 1.14 |
| P14148 | **60S ribosomal protein L7 OS=Mus musculus GN=Rpl7 PE=1 SV=2** | 172094.8 | 150854.7 | 1.14 |
| O35841 | **Apoptosis inhibitor 5 OS=Mus musculus GN=Api5 PE=1 SV=2** | 10528.3 | 9193.9 | 1.15 |
| Q9D7S7 | **60S ribosomal protein L22-like 1 OS=Mus musculus GN=Rpl22l1 PE=1 SV=1** | 12122.4 | 10583.7 | 1.15 |
| P54728 | **UV excision repair protein RAD23 homolog B OS=Mus musculus GN=Rad23b PE=1 SV=2** | 195378.6 | 170102.4 | 1.15 |
| Q3THS6 | **S-adenosylmethionine synthase isoform type-2 OS=Mus musculus GN=Mat2a PE=2 SV=2** | 22700.9 | 19748.0 | 1.15 |
| Q9CQ60 | **6-phosphogluconolactonase OS=Mus musculus GN=Pgls PE=2 SV=1** | 140594.4 | 121916.7 | 1.15 |
| Q61792 | **LIM and SH3 domain protein 1 OS=Mus musculus GN=Lasp1 PE=1 SV=1** | 249942.8 | 216670.5 | 1.15 |
| O55135 | **Eukaryotic translation initiation factor 6 OS=Mus musculus GN=Eif6 PE=1 SV=2** | 347084.7 | 300726.4 | 1.15 |
| P68369 | **Tubulin alpha-1A chain OS=Mus musculus GN=Tuba1a PE=1 SV=1** | 14110.6 | 12191.9 | 1.16 |
| P80316 | **T-complex protein 1 subunit epsilon OS=Mus musculus GN=Cct5 PE=1 SV=1** | 159506.1 | 137781.2 | 1.16 |
| Q9CXL3 | **Uncharacterized protein C7orf50 homolog OS=Mus musculus PE=1 SV=3** | 32150.8 | 27695.8 | 1.16 |
| P27661 | **Histone H2AX OS=Mus musculus GN=H2afx PE=1 SV=2** | 148664.2 | 127794.0 | 1.16 |
| P34022 | **Ran-specific GTPase-activating protein OS=Mus musculus GN=Ranbp1 PE=1 SV=2** | 907768.9 | 780126.5 | 1.16 |
| Q64727 | **Vinculin OS=Mus musculus GN=Vcl PE=1 SV=4** | 772586.9 | 662152.8 | 1.17 |
| Q8VDM4 | **26S proteasome non-ATPase regulatory subunit 2 OS=Mus musculus GN=Psmd2 PE=1 SV=1** | 100716.5 | 85700.4 | 1.18 |
| P14206 | **40S ribosomal protein SA OS=Mus musculus GN=Rpsa PE=1 SV=4** | 1565615.4 | 1331765.8 | 1.18 |
| Q9CZD3 | **Glycine--tRNA ligase OS=Mus musculus GN=Gars PE=1 SV=1** | 125999.7 | 107075.8 | 1.18 |
| O70133 | **ATP-dependent RNA helicase A OS=Mus musculus GN=Dhx9 PE=1 SV=2** | 46966.9 | 39905.0 | 1.18 |
| Q9R0Y5 | **Adenylate kinase isoenzyme 1 OS=Mus musculus GN=Ak1 PE=1 SV=1** | 31212.6 | 26458.6 | 1.18 |
| Q9CPU0 | **Lactoylglutathione lyase OS=Mus musculus GN=Glo1 PE=1 SV=3** | 675707.9 | 571693.8 | 1.18 |
| Q8BG05 | **Heterogeneous nuclear ribonucleoprotein A3 OS=Mus musculus GN=Hnrnpa3 PE=1 SV=1** | 1156390.1 | 978152.2 | 1.18 |
| Q9Z1Q9 | **Valine--tRNA ligase OS=Mus musculus GN=Vars PE=2 SV=1** | 115943.5 | 98055.7 | 1.18 |
| Q8R016 | **Bleomycin hydrolase OS=Mus musculus GN=Blmh PE=1 SV=1** | 100161.7 | 84646.7 | 1.18 |

| | | | | |
|---|---|---|---|---|
| Q61024 | **Asparagine synthetase [glutamine-hydrolyzing] OS=Mus musculus GN=Asns PE=2 SV=3** | 33337.7 | 27981.3 | 1.19 |
| O35639 | **Annexin A3 OS=Mus musculus GN=Anxa3 PE=1 SV=4** | 53092.7 | 44505.5 | 1.19 |
| P42932 | **T-complex protein 1 subunit theta OS=Mus musculus GN=Cct8 PE=1 SV=3** | 479394.5 | 401725.3 | 1.19 |
| Q05D44 | **Eukaryotic translation initiation factor 5B OS=Mus musculus GN=Eif5b PE=1 SV=2** | 22032.2 | 18422.1 | 1.20 |
| Q8R010 | **Aminoacyl tRNA synthase complex-interacting multifunctional protein 2 OS=Mus musculus GN=Aimp2 PE=1 SV=2** | 21271.6 | 17740.2 | 1.20 |
| O54879 | **High mobility group protein B3 OS=Mus musculus GN=Hmgb3 PE=2 SV=3** | 38763.9 | 32315.7 | 1.20 |
| P51410 | **60S ribosomal protein L9 OS=Mus musculus GN=Rpl9 PE=1 SV=2** | 16643.5 | 13835.7 | 1.20 |
| P20029 | **78 kDa glucose-regulated protein OS=Mus musculus GN=Hspa5 PE=1 SV=3** | 790739.0 | 656534.4 | 1.20 |
| P31786 | **Acyl-CoA-binding protein OS=Mus musculus GN=Dbi PE=1 SV=2** | 901059.6 | 746552.4 | 1.21 |
| O88342 | **WD repeat-containing protein 1 OS=Mus musculus GN=Wdr1 PE=1 SV=3** | 81889.2 | 67756.8 | 1.21 |
| P50580 | **Proliferation-associated protein 2G4 OS=Mus musculus GN=Pa2g4 PE=1 SV=3** | 1210522.6 | 996185.7 | 1.22 |
| Q9R1T2 | **SUMO-activating enzyme subunit 1 OS=Mus musculus GN=Sae1 PE=1 SV=1** | 5365.6 | 4409.5 | 1.22 |
| Q91VK1 | **Basic leucine zipper and W2 domain-containing protein 2 OS=Mus musculus GN=Bzw2 PE=1 SV=1** | 49972.4 | 41004.8 | 1.22 |
| O35737 | **Heterogeneous nuclear ribonucleoprotein H OS=Mus musculus GN=Hnrnph1 PE=1 SV=3** | 174497.0 | 142752.1 | 1.22 |
| Q91YR9 | **Prostaglandin reductase 1 OS=Mus musculus GN=Ptgr1 PE=1 SV=2** | 66359.4 | 54095.0 | 1.23 |
| P10126 | **Elongation factor 1-alpha 1 OS=Mus musculus GN=Eef1a1 PE=1 SV=3** | 2595210.4 | 2112553.0 | 1.23 |
| Q9JJI8 | **60S ribosomal protein L38 OS=Mus musculus GN=Rpl38 PE=3 SV=3** | 58860.2 | 47868.3 | 1.23 |
| Q3U1J4 | **DNA damage-binding protein 1 OS=Mus musculus GN=Ddb1 PE=1 SV=2** | 384919.0 | 311646.1 | 1.24 |
| P49722 | **Proteasome subunit alpha type-2 OS=Mus musculus GN=Psma2 PE=1 SV=3** | 353056.3 | 285110.5 | 1.24 |
| P05064 | **Fructose-bisphosphate aldolase A OS=Mus musculus GN=Aldoa PE=1 SV=2** | 5237860.7 | 4228531.0 | 1.24 |
| Q5RI75 | **Ras and EF-hand domain-containing protein homolog OS=Mus musculus GN=Rasef PE=2 SV=1** | 38390.6 | 30942.5 | 1.24 |
| Q07813 | **Apoptosis regulator BAX OS=Mus musculus GN=Bax PE=1 SV=1** | 49928.5 | 40048.4 | 1.25 |
| Q61316 | **Heat shock 70 kDa protein 4 OS=Mus musculus GN=Hspa4 PE=1 SV=1** | 1358143.8 | 1087625.2 | 1.25 |
| P62960 | **Nuclease-sensitive element-binding protein 1 OS=Mus musculus GN=Ybx1 PE=1 SV=3** | 787283.8 | 630051.6 | 1.25 |
| P27048 | **Small nuclear ribonucleoprotein-associated protein B OS=Mus musculus GN=Snrpb PE=1 SV=1** | 20637.6 | 16499.8 | 1.25 |
| P99024 | **Tubulin beta-5 chain OS=Mus musculus GN=Tubb5 PE=1 SV=1** | 327944.7 | 261403.7 | 1.25 |
| P63038 | **60 kDa heat shock protein, mitochondrial OS=Mus musculus GN=Hspd1 PE=1 SV=1** | 246425.4 | 196372.7 | 1.25 |
| Q91YE6 | **Importin-9 OS=Mus musculus GN=Ipo9 PE=1 SV=3** | 31982.7 | 25481.6 | 1.26 |
| P19096 | **Fatty acid synthase OS=Mus musculus GN=Fasn PE=1 SV=2** | 707054.0 | 563307.6 | 1.26 |

| | | | | |
|---|---|---|---|---|
| Q9CZX8 | **40S ribosomal protein S19 OS=Mus musculus GN=Rps19 PE=1 SV=3** | 65067.9 | 51797.6 | 1.26 |
| Q9D8W5 | **26S proteasome non-ATPase regulatory subunit 12 OS=Mus musculus GN=Psmd12 PE=1 SV=4** | 55389.6 | 44043.2 | 1.26 |
| O88844 | **Isocitrate dehydrogenase [NADP] cytoplasmic OS=Mus musculus GN=Idh1 PE=1 SV=2** | 55949.1 | 44277.8 | 1.26 |
| P62315 | **Small nuclear ribonucleoprotein Sm D1 OS=Mus musculus GN=Snrpd1 PE=3 SV=1** | 67363.3 | 53159.6 | 1.27 |
| Q9Z1Q5 | **Chloride intracellular channel protein 1 OS=Mus musculus GN=Clic1 PE=1 SV=3** | 176750.8 | 139263.3 | 1.27 |
| Q8K2B3 | **Succinate dehydrogenase [ubiquinone] flavoprotein subunit, mitochondrial OS=Mus musculus GN=Sdha PE=1 SV=1** | 10901.9 | 8585.0 | 1.27 |
| P27659 | **60S ribosomal protein L3 OS=Mus musculus GN=Rpl3 PE=1 SV=3** | 402805.2 | 316757.2 | 1.27 |
| P62305 | **Small nuclear ribonucleoprotein E OS=Mus musculus GN=Snrpe PE=3 SV=1** | 86088.6 | 67547.3 | 1.27 |
| P47911 | **60S ribosomal protein L6 OS=Mus musculus GN=Rpl6 PE=1 SV=3** | 554572.6 | 434716.6 | 1.28 |
| Q6PIP5 | **NudC domain-containing protein 1 OS=Mus musculus GN=Nudcd1 PE=2 SV=2** | 199819.7 | 156499.9 | 1.28 |
| P80314 | **T-complex protein 1 subunit beta OS=Mus musculus GN=Cct2 PE=1 SV=4** | 1008761.7 | 789880.6 | 1.28 |
| P35980 | **60S ribosomal protein L18 OS=Mus musculus GN=Rpl18 PE=2 SV=3** | 32879.9 | 25689.2 | 1.28 |
| Q64433 | **10 kDa heat shock protein, mitochondrial OS=Mus musculus GN=Hspe1 PE=1 SV=2** | 307013.2 | 239799.8 | 1.28 |
| Q80X50 | **Ubiquitin-associated protein 2-like OS=Mus musculus GN=Ubap2l PE=1 SV=1** | 21588.9 | 16826.4 | 1.28 |
| Q9EPL8 | **Importin-7 OS=Mus musculus GN=Ipo7 PE=1 SV=2** | 79142.4 | 61622.3 | 1.28 |
| P70168 | **Importin subunit beta-1 OS=Mus musculus GN=Kpnb1 PE=1 SV=2** | 639824.6 | 497804.2 | 1.29 |
| Q9CZ44 | **NSFL1 cofactor p47 OS=Mus musculus GN=Nsfl1c PE=1 SV=1** | 526449.8 | 408740.5 | 1.29 |
| Q91V92 | **ATP-citrate synthase OS=Mus musculus GN=Acly PE=1 SV=1** | 146256.8 | 113455.2 | 1.29 |
| P97372 | **Proteasome activator complex subunit 2 OS=Mus musculus GN=Psme2 PE=2 SV=4** | 73219.5 | 56771.5 | 1.29 |
| P14211 | **Calreticulin OS=Mus musculus GN=Calr PE=1 SV=1** | 1645270.7 | 1268424.9 | 1.30 |
| P53026 | **60S ribosomal protein L10a OS=Mus musculus GN=Rpl10a PE=1 SV=3** | 31960.1 | 24603.9 | 1.30 |
| Q9R1P4 | **Proteasome subunit alpha type-1 OS=Mus musculus GN=Psma1 PE=1 SV=1** | 106073.7 | 81548.7 | 1.30 |
| Q923D2 | **Flavin reductase (NADPH) OS=Mus musculus GN=Blvrb PE=2 SV=3** | 240220.8 | 184444.2 | 1.30 |
| Q9Z204 | **Heterogeneous nuclear ribonucleoproteins C1/C2 OS=Mus musculus GN=Hnrnpc PE=1 SV=1** | 177045.0 | 135758.7 | 1.30 |
| P11983 | **T-complex protein 1 subunit alpha OS=Mus musculus GN=Tcp1 PE=1 SV=3** | 390795.5 | 298120.5 | 1.31 |
| A2AJK6 | **Chromodomain-helicase-DNA-binding protein 7 OS=Mus musculus GN=Chd7 PE=1 SV=1** | 7267.3 | 5534.7 | 1.31 |
| O55234 | **Proteasome subunit beta type-5 OS=Mus musculus GN=Psmb5 PE=1 SV=3** | 401614.7 | 304755.5 | 1.32 |
| Q9CWI3 | **BRCA2 and CDKN1A-interacting protein OS=Mus musculus GN=Bccip PE=2 SV=1** | 90307.5 | 68485.9 | 1.32 |
| P42208 | **Septin-2 OS=Mus musculus GN=Sept2 PE=1 SV=2** | 82765.6 | 62713.7 | 1.32 |
| P08030 | **Adenine phosphoribosyltransferase OS=Mus musculus GN=Aprt PE=1 SV=2** | 100323.6 | 76008.0 | 1.32 |

| | | | | |
|---|---|---|---|---|
| Q8JZQ9 | **Eukaryotic translation initiation factor 3 subunit B OS=Mus musculus GN=Eif3b PE=1 SV=1** | 262241.4 | 198503.3 | 1.32 |
| P61514 | **60S ribosomal protein L37a OS=Mus musculus GN=Rpl37a PE=3 SV=2** | 33894.1 | 25592.3 | 1.32 |
| Q9ERK4 | **Exportin-2 OS=Mus musculus GN=Cse1l PE=2 SV=1** | 73498.9 | 55433.8 | 1.33 |
| P21550 | **Beta-enolase OS=Mus musculus GN=Eno3 PE=1 SV=3** | 91844.3 | 69193.9 | 1.33 |
| Q9D0R2 | **Threonine--tRNA ligase, cytoplasmic OS=Mus musculus GN=Tars PE=1 SV=2** | 423988.3 | 319377.3 | 1.33 |
| Q62167 | **ATP-dependent RNA helicase DDX3X OS=Mus musculus GN=Ddx3x PE=1 SV=3** | 27103.6 | 20396.3 | 1.33 |
| Q91VW3 | **SH3 domain-binding glutamic acid-rich-like protein 3 OS=Mus musculus GN=Sh3bgrl3 PE=1 SV=1** | 94342.7 | 70954.5 | 1.33 |
| Q9Z2Y8 | **Proline synthase co-transcribed bacterial homolog protein OS=Mus musculus GN=Prosc PE=1 SV=1** | 19795.1 | 14878.8 | 1.33 |
| Q8BP67 | **60S ribosomal protein L24 OS=Mus musculus GN=Rpl24 PE=1 SV=2** | 149847.1 | 111497.3 | 1.34 |
| P17182 | **Alpha-enolase OS=Mus musculus GN=Eno1 PE=1 SV=3** | 12346618.0 | 9186588.5 | 1.34 |
| P63323 | **40S ribosomal protein S12 OS=Mus musculus GN=Rps12 PE=1 SV=2** | 500285.1 | 371993.7 | 1.34 |
| P56812 | **Programmed cell death protein 5 OS=Mus musculus GN=Pdcd5 PE=1 SV=3** | 309133.2 | 229222.8 | 1.35 |
| P62259 | **14-3-3 protein epsilon OS=Mus musculus GN=Ywhae PE=1 SV=1** | 4209348.2 | 3119414.2 | 1.35 |
| Q62418 | **Drebrin-like protein OS=Mus musculus GN=Dbnl PE=1 SV=2** | 39335.7 | 29130.0 | 1.35 |
| Q99K51 | **Plastin-3 OS=Mus musculus GN=Pls3 PE=1 SV=3** | 39798.3 | 29463.4 | 1.35 |
| P05202 | **Aspartate aminotransferase, mitochondrial OS=Mus musculus GN=Got2 PE=1 SV=1** | 282625.8 | 209124.2 | 1.35 |
| P80315 | **T-complex protein 1 subunit delta OS=Mus musculus GN=Cct4 PE=1 SV=3** | 172154.2 | 127152.4 | 1.35 |
| O88544 | **COP9 signalosome complex subunit 4 OS=Mus musculus GN=Cops4 PE=1 SV=1** | 25607.0 | 18806.8 | 1.36 |
| Q922F4 | **Tubulin beta-6 chain OS=Mus musculus GN=Tubb6 PE=1 SV=1** | 87706.7 | 64272.6 | 1.36 |
| P70697 | **Uroporphyrinogen decarboxylase OS=Mus musculus GN=Urod PE=1 SV=2** | 16127.4 | 11817.0 | 1.36 |
| P45376 | **Aldose reductase OS=Mus musculus GN=Akr1b1 PE=1 SV=3** | 379843.8 | 278233.4 | 1.37 |
| P62334 | **26S protease regulatory subunit 10B OS=Mus musculus GN=Psmc6 PE=1 SV=1** | 42020.9 | 30739.8 | 1.37 |
| Q61937 | **Nucleophosmin OS=Mus musculus GN=Npm1 PE=1 SV=1** | 3833606.0 | 2800523.4 | 1.37 |
| P97351 | **40S ribosomal protein S3a OS=Mus musculus GN=Rps3a PE=1 SV=3** | 972998.2 | 710377.7 | 1.37 |
| Q91YP2 | **Neurolysin, mitochondrial OS=Mus musculus GN=Nln PE=2 SV=1** | 6286.4 | 4579.5 | 1.37 |
| P97371 | **Proteasome activator complex subunit 1 OS=Mus musculus GN=Psme1 PE=2 SV=2** | 32821.5 | 23852.8 | 1.38 |
| Q99J09 | **Methylosome protein 50 OS=Mus musculus GN=Wdr77 PE=1 SV=1** | 39028.6 | 28346.9 | 1.38 |
| Q922R8 | **Protein disulfide-isomerase A6 OS=Mus musculus GN=Pdia6 PE=1 SV=3** | 96198.6 | 69868.8 | 1.38 |
| P08228 | **Superoxide dismutase [Cu-Zn] OS=Mus musculus GN=Sod1 PE=1 SV=2** | 4188006.3 | 3040473.6 | 1.38 |
| P26638 | **Serine--tRNA ligase, cytoplasmic OS=Mus musculus GN=Sars PE=2 SV=3** | 17532.8 | 12701.8 | 1.38 |
| Q8BGD8 | **Cytochrome c oxidase assembly factor 6 homolog OS=Mus musculus GN=Coa6 PE=3 SV=1** | 3487.0 | 2522.3 | 1.38 |

| | | | | |
|---|---|---|---|---|
| Q02819 | **Nucleobindin-1 OS=Mus musculus GN=Nucb1 PE=1 SV=2** | 226832.0 | 163258.2 | 1.39 |
| P28352 | **DNA-(apurinic or apyrimidinic site) lyase OS=Mus musculus GN=Apex1 PE=1 SV=2** | 812447.7 | 583819.8 | 1.39 |
| P47962 | **60S ribosomal protein L5 OS=Mus musculus GN=Rpl5 PE=1 SV=3** | 1468504.1 | 1051960.7 | 1.40 |
| Q9D727 | **Uncharacterized protein C6orf226 homolog OS=Mus musculus PE=4 SV=1** | 19844.7 | 14213.6 | 1.40 |
| P62301 | **40S ribosomal protein S13 OS=Mus musculus GN=Rps13 PE=1 SV=2** | 80144.3 | 57381.4 | 1.40 |
| Q6ZWV7 | **60S ribosomal protein L35 OS=Mus musculus GN=Rpl35 PE=2 SV=1** | 69392.0 | 49680.3 | 1.40 |
| Q91VI7 | **Ribonuclease inhibitor OS=Mus musculus GN=Rnh1 PE=1 SV=1** | 1103811.9 | 787296.3 | 1.40 |
| P07091 | **Protein S100-A4 OS=Mus musculus GN=S100a4 PE=1 SV=1** | 350510.6 | 249193.7 | 1.41 |
| O09167 | **60S ribosomal protein L21 OS=Mus musculus GN=Rpl21 PE=2 SV=3** | 335028.2 | 238108.7 | 1.41 |
| Q8BGB7 | **Enolase-phosphatase E1 OS=Mus musculus GN=Enoph1 PE=2 SV=1** | 101758.5 | 72267.5 | 1.41 |
| Q9CQE8 | **UPF0568 protein C14orf166 homolog OS=Mus musculus PE=2 SV=1** | 26065.0 | 18507.4 | 1.41 |
| Q99LI5 | **Zinc finger protein 281 OS=Mus musculus GN=Znf281 PE=1 SV=1** | 20638.8 | 14639.5 | 1.41 |
| P60843 | **Eukaryotic initiation factor 4A-I OS=Mus musculus GN=Eif4a1 PE=1 SV=1** | 467690.7 | 330882.0 | 1.41 |
| P06797 | **Cathepsin L1 OS=Mus musculus GN=Ctsl PE=1 SV=2** | 177334.4 | 125081.2 | 1.42 |
| Q9D819 | **Inorganic pyrophosphatase OS=Mus musculus GN=Ppa1 PE=1 SV=1** | 133264.5 | 93956.7 | 1.42 |
| Q6P1B1 | **Xaa-Pro aminopeptidase 1 OS=Mus musculus GN=Xpnpep1 PE=2 SV=1** | 75266.3 | 53016.6 | 1.42 |
| Q60631 | **Growth factor receptor-bound protein 2 OS=Mus musculus GN=Grb2 PE=1 SV=1** | 34137.9 | 24006.3 | 1.42 |
| Q99PT1 | **Rho GDP-dissociation inhibitor 1 OS=Mus musculus GN=Arhgdia PE=1 SV=3** | 1625225.7 | 1141097.5 | 1.42 |
| P62500 | **TSC22 domain family protein 1 OS=Mus musculus GN=Tsc22d1 PE=1 SV=2** | 12531.7 | 8789.1 | 1.43 |
| O09061 | **Proteasome subunit beta type-1 OS=Mus musculus GN=Psmb1 PE=1 SV=1** | 306696.7 | 215089.1 | 1.43 |
| Q91VX2 | **Ubiquitin-associated protein 2 OS=Mus musculus GN=Ubap2 PE=1 SV=1** | 5046.0 | 3538.5 | 1.43 |
| P56480 | **ATP synthase subunit beta, mitochondrial OS=Mus musculus GN=Atp5b PE=1 SV=2** | 206748.4 | 144870.0 | 1.43 |
| Q9D0F9 | **Phosphoglucomutase-1 OS=Mus musculus GN=Pgm1 PE=1 SV=4** | 80600.1 | 56425.5 | 1.43 |
| P14685 | **26S proteasome non-ATPase regulatory subunit 3 OS=Mus musculus GN=Psmd3 PE=1 SV=3** | 61634.7 | 43133.8 | 1.43 |
| P47963 | **60S ribosomal protein L13 OS=Mus musculus GN=Rpl13 PE=2 SV=3** | 67533.1 | 47119.4 | 1.43 |
| Q64437 | **Alcohol dehydrogenase class 4 mu/sigma chain OS=Mus musculus GN=Adh7 PE=2 SV=2** | 28921.5 | 20159.3 | 1.43 |
| Q9R1P1 | **Proteasome subunit beta type-3 OS=Mus musculus GN=Psmb3 PE=1 SV=1** | 614178.0 | 426761.9 | 1.44 |
| P16858 | **Glyceraldehyde-3-phosphate dehydrogenase OS=Mus musculus GN=Gapdh PE=1 SV=2** | 2754591.3 | 1909097.8 | 1.44 |
| P17426 | **AP-2 complex subunit alpha-1 OS=Mus musculus GN=Ap2a1 PE=1 SV=1** | 23045.4 | 15962.2 | 1.44 |

| | | | | |
|---|---|---|---|---|
| P46664 | **Adenylosuccinate synthetase isozyme 2 OS=Mus musculus GN=Adss PE=1 SV=2** | 393895.7 | 272404.1 | 1.45 |
| Q64674 | **Spermidine synthase OS=Mus musculus GN=Srm PE=2 SV=1** | 288137.4 | 199137.5 | 1.45 |
| P97315 | **Cysteine and glycine-rich protein 1 OS=Mus musculus GN=Csrp1 PE=1 SV=3** | 3907.6 | 2694.1 | 1.45 |
| P62962 | **Profilin-1 OS=Mus musculus GN=Pfn1 PE=1 SV=2** | 1996225.3 | 1372200.1 | 1.45 |
| P30681 | **High mobility group protein B2 OS=Mus musculus GN=Hmgb2 PE=1 SV=3** | 418308.2 | 287537.9 | 1.45 |
| Q9QUR6 | **Prolyl endopeptidase OS=Mus musculus GN=Prep PE=2 SV=1** | 828084.0 | 569193.3 | 1.45 |
| Q9QUM9 | **Proteasome subunit alpha type-6 OS=Mus musculus GN=Psma6 PE=1 SV=1** | 607504.3 | 417299.4 | 1.46 |
| Q9Z2U1 | **Proteasome subunit alpha type-5 OS=Mus musculus GN=Psma5 PE=1 SV=1** | 826449.3 | 567336.9 | 1.46 |
| O08583 | **THO complex subunit 4 OS=Mus musculus GN=Alyref PE=1 SV=3** | 445038.8 | 304555.4 | 1.46 |
| Q7TQI3 | **Ubiquitin thioesterase OTUB1 OS=Mus musculus GN=Otub1 PE=1 SV=2** | 100529.3 | 68617.2 | 1.47 |
| Q6IRU5 | **Clathrin light chain B OS=Mus musculus GN=Cltb PE=1 SV=1** | 38937.4 | 26525.8 | 1.47 |
| O89086 | **RNA-binding protein 3 OS=Mus musculus GN=Rbm3 PE=2 SV=1** | 901258.2 | 613787.5 | 1.47 |
| Q8BGQ7 | **Alanine--tRNA ligase, cytoplasmic OS=Mus musculus GN=Aars PE=1 SV=1** | 67190.0 | 45689.1 | 1.47 |
| P60122 | **RuvB-like 1 OS=Mus musculus GN=Ruvbl1 PE=1 SV=1** | 114892.8 | 77877.0 | 1.48 |
| P62245 | **40S ribosomal protein S15a OS=Mus musculus GN=Rps15a PE=1 SV=2** | 214732.8 | 145445.4 | 1.48 |
| O08553 | **Dihydropyrimidinase-related protein 2 OS=Mus musculus GN=Dpysl2 PE=1 SV=2** | 721841.5 | 488578.0 | 1.48 |
| Q8K183 | **Pyridoxal kinase OS=Mus musculus GN=Pdxk PE=1 SV=1** | 26223.2 | 17723.6 | 1.48 |
| P56380 | **Bis(5'-nucleosyl)-tetraphosphatase [asymmetrical] OS=Mus musculus GN=Nudt2 PE=1 SV=3** | 36309.1 | 24513.9 | 1.48 |
| P20152 | **Vimentin OS=Mus musculus GN=Vim PE=1 SV=3** | 40677506.4 | 27407058.3 | 1.48 |
| Q3UM45 | **Protein phosphatase 1 regulatory subunit 7 OS=Mus musculus GN=Ppp1r7 PE=1 SV=2** | 13335.5 | 8965.7 | 1.49 |
| P99026 | **Proteasome subunit beta type-4 OS=Mus musculus GN=Psmb4 PE=1 SV=1** | 446045.1 | 299397.6 | 1.49 |
| Q9JHU4 | **Cytoplasmic dynein 1 heavy chain 1 OS=Mus musculus GN=Dync1h1 PE=1 SV=2** | 10125.5 | 6771.1 | 1.50 |
| Q9WTM5 | **RuvB-like 2 OS=Mus musculus GN=Ruvbl2 PE=2 SV=3** | 91585.6 | 61213.9 | 1.50 |
| P21460 | **Cystatin-C OS=Mus musculus GN=Cst3 PE=2 SV=2** | 84925.4 | 56749.1 | 1.50 |
| Q99JI4 | **26S proteasome non-ATPase regulatory subunit 6 OS=Mus musculus GN=Psmd6 PE=1 SV=1** | 56216.6 | 37148.4 | 1.51 |
| P10518 | **Delta-aminolevulinic acid dehydratase OS=Mus musculus GN=Alad PE=1 SV=1** | 218973.7 | 144473.1 | 1.52 |
| P47955 | **60S acidic ribosomal protein P1 OS=Mus musculus GN=Rplp1 PE=3 SV=1** | 685391.4 | 451258.5 | 1.52 |
| P62889 | **60S ribosomal protein L30 OS=Mus musculus GN=Rpl30 PE=3 SV=2** | 536279.8 | 352443.3 | 1.52 |
| Q61171 | **Peroxiredoxin-2 OS=Mus musculus GN=Prdx2 PE=1 SV=3** | 51921.4 | 34116.2 | 1.52 |
| Q9CR57 | **60S ribosomal protein L14 OS=Mus musculus GN=Rpl14 PE=1 SV=3** | 53854.5 | 35348.9 | 1.52 |
| Q8VCT3 | **Aminopeptidase B OS=Mus musculus GN=Rnpep PE=2 SV=2** | 21106.8 | 13826.7 | 1.53 |
| Q9R1P3 | **Proteasome subunit beta type-2 OS=Mus musculus GN=Psmb2 PE=1 SV=1** | 543655.2 | 355808.0 | 1.53 |

| | | | | |
|---|---|---|---|---|
| Q9ESY9 | **Gamma-interferon-inducible lysosomal thiol reductase OS=Mus musculus GN=Ifi30 PE=1 SV=3** | 8908.7 | 5818.3 | 1.53 |
| Q01768 | **Nucleoside diphosphate kinase B OS=Mus musculus GN=Nme2 PE=1 SV=1** | 2626135.4 | 1712697.3 | 1.53 |
| Q9D0B6 | **Protein PBDC1 OS=Mus musculus GN=Pbdc1 PE=2 SV=1** | 32444.1 | 21097.6 | 1.54 |
| P23591 | **GDP-L-fucose synthase OS=Mus musculus GN=Tsta3 PE=2 SV=3** | 2943.2 | 1913.0 | 1.54 |
| Q9WUA2 | **Phenylalanine--tRNA ligase beta subunit OS=Mus musculus GN=Farsb PE=2 SV=2** | 8268.8 | 5356.0 | 1.54 |
| Q9Z2U0 | **Proteasome subunit alpha type-7 OS=Mus musculus GN=Psma7 PE=1 SV=1** | 597025.2 | 383944.9 | 1.55 |
| Q9JK81 | **UPF0160 protein MYG1, mitochondrial OS=Mus musculus GN=Myg1 PE=1 SV=1** | 33152.9 | 21294.6 | 1.56 |
| Q9CZU6 | **Citrate synthase, mitochondrial OS=Mus musculus GN=Cs PE=1 SV=1** | 7352.4 | 4716.9 | 1.56 |
| P61957 | **Small ubiquitin-related modifier 2 OS=Mus musculus GN=Sumo2 PE=1 SV=1** | 486401.9 | 310307.4 | 1.57 |
| Q8VI75 | **Importin-4 OS=Mus musculus GN=Ipo4 PE=1 SV=1** | 10090.6 | 6433.6 | 1.57 |
| P08207 | **Protein S100-A10 OS=Mus musculus GN=S100a10 PE=1 SV=2** | 158760.4 | 100223.4 | 1.58 |
| Q62426 | **Cystatin-B OS=Mus musculus GN=Cstb PE=2 SV=1** | 180621.7 | 113894.2 | 1.59 |
| Q62193 | **Replication protein A 32 kDa subunit OS=Mus musculus GN=Rpa2 PE=1 SV=1** | 13747.4 | 8556.9 | 1.61 |
| P56212 | **cAMP-regulated phosphoprotein 19 OS=Mus musculus GN=Arpp19 PE=1 SV=2** | 12523.7 | 7783.3 | 1.61 |
| Q9CQ48 | **NudC domain-containing protein 2 OS=Mus musculus GN=Nudcd2 PE=1 SV=1** | 9969.1 | 6190.7 | 1.61 |
| O70310 | **Glycylpeptide N-tetradecanoyltransferase 1 OS=Mus musculus GN=Nmt1 PE=1 SV=1** | 50091.2 | 31090.3 | 1.61 |
| Q9JII6 | **Alcohol dehydrogenase [NADP(+)] OS=Mus musculus GN=Akr1a1 PE=1 SV=3** | 216929.0 | 133541.7 | 1.62 |
| Q9EST5 | **Acidic leucine-rich nuclear phosphoprotein 32 family member B OS=Mus musculus GN=Anp32b PE=1 SV=1** | 452701.2 | 277728.6 | 1.63 |
| P47754 | **F-actin-capping protein subunit alpha-2 OS=Mus musculus GN=Capza2 PE=1 SV=3** | 101444.3 | 62152.6 | 1.63 |
| P62918 | **60S ribosomal protein L8 OS=Mus musculus GN=Rpl8 PE=1 SV=2** | 208003.4 | 127023.5 | 1.64 |
| Q5SUR0 | **Phosphoribosylformylglycinamidine synthase OS=Mus musculus GN=Pfas PE=2 SV=1** | 38974.4 | 23786.9 | 1.64 |
| Q01730 | **Ras suppressor protein 1 OS=Mus musculus GN=Rsu1 PE=2 SV=3** | 80692.7 | 49182.7 | 1.64 |
| P62192 | **26S protease regulatory subunit 4 OS=Mus musculus GN=Psmc1 PE=1 SV=1** | 12354.1 | 7502.9 | 1.65 |
| Q9DBJ1 | **Phosphoglycerate mutase 1 OS=Mus musculus GN=Pgam1 PE=1 SV=3** | 960120.4 | 580603.2 | 1.65 |
| O88569 | **Heterogeneous nuclear ribonucleoproteins A2/B1 OS=Mus musculus GN=Hnrnpa2b1 PE=1 SV=2** | 1340258.3 | 805691.4 | 1.66 |
| Q8C1B7 | **Septin-11 OS=Mus musculus GN=Sept11 PE=1 SV=4** | 18534.9 | 10943.8 | 1.69 |
| P63094 | **Guanine nucleotide-binding protein G(s) subunit alpha isoforms short OS=Mus musculus GN=Gnas PE=1 SV=1** | 25429.4 | 14992.7 | 1.70 |
| P07141 | **Macrophage colony-stimulating factor 1 OS=Mus musculus GN=Csf1 PE=1 SV=2** | 27917.7 | 16425.1 | 1.70 |
| P07356 | **Annexin A2 OS=Mus musculus GN=Anxa2 PE=1 SV=2** | 1137028.4 | 668886.3 | 1.70 |
| Q9WU78 | **Programmed cell death 6-interacting protein OS=Mus musculus GN=Pdcd6ip PE=1 SV=3** | 27091.3 | 15883.7 | 1.71 |
| Q9JIF7 | **Coatomer subunit beta OS=Mus musculus GN=Copb1 PE=1 SV=1** | 6703.4 | 3926.5 | 1.71 |

| | | | | |
|---|---|---|---|---|
| Q9CPR4 | **60S ribosomal protein L17 OS=Mus musculus GN=Rpl17 PE=2 SV=3** | 56913.9 | 33324.8 | 1.71 |
| P12970 | **60S ribosomal protein L7a OS=Mus musculus GN=Rpl7a PE=1 SV=2** | 568312.2 | 332464.9 | 1.71 |
| P10493 | **Nidogen-1 OS=Mus musculus GN=Nid1 PE=1 SV=2** | 8423.5 | 4885.6 | 1.72 |
| Q8CIG8 | **Protein arginine N-methyltransferase 5 OS=Mus musculus GN=Prmt5 PE=1 SV=3** | 16361.6 | 9488.6 | 1.72 |
| Q4VAA2 | **Protein CDV3 OS=Mus musculus GN=Cdv3 PE=1 SV=2** | 378341.0 | 219059.9 | 1.73 |
| P31324 | **cAMP-dependent protein kinase type II-beta regulatory subunit OS=Mus musculus GN=Prkar2b PE=1 SV=3** | 11854.7 | 6856.0 | 1.73 |
| P16254 | **Signal recognition particle 14 kDa protein OS=Mus musculus GN=Srp14 PE=1 SV=1** | 105884.1 | 60684.2 | 1.74 |
| Q99MN1 | **Lysine--tRNA ligase OS=Mus musculus GN=Kars PE=1 SV=1** | 76698.9 | 43956.3 | 1.74 |
| P52293 | **Importin subunit alpha-1 OS=Mus musculus GN=Kpna2 PE=1 SV=2** | 224372.1 | 128209.0 | 1.75 |
| Q61595 | **Kinectin OS=Mus musculus GN=Ktn1 PE=2 SV=1** | 20610.2 | 11770.9 | 1.75 |
| O35658 | **Complement component 1 Q subcomponent-binding protein, mitochondrial OS=Mus musculus GN=C1qbp PE=1 SV=1** | 231262.2 | 131792.5 | 1.75 |
| P08249 | **Malate dehydrogenase, mitochondrial OS=Mus musculus GN=Mdh2 PE=1 SV=3** | 2435239.9 | 1372419.6 | 1.77 |
| P17751 | **Triosephosphate isomerase OS=Mus musculus GN=Tpi1 PE=1 SV=4** | 4705802.4 | 2632216.5 | 1.79 |
| P21107 | **Tropomyosin alpha-3 chain OS=Mus musculus GN=Tpm3 PE=2 SV=3** | 1559126.2 | 871781.9 | 1.79 |
| O35143 | **ATPase inhibitor, mitochondrial OS=Mus musculus GN=Atpif1 PE=1 SV=2** | 9898.1 | 5458.4 | 1.81 |
| O08784 | **Treacle protein OS=Mus musculus GN=Tcof1 PE=1 SV=1** | 11810.8 | 6498.0 | 1.82 |
| P14869 | **60S acidic ribosomal protein P0 OS=Mus musculus GN=Rplp0 PE=1 SV=3** | 157428.2 | 86492.9 | 1.82 |
| P09405 | **Nucleolin OS=Mus musculus GN=Ncl PE=1 SV=2** | 4290089.1 | 2356014.8 | 1.82 |
| P14115 | **60S ribosomal protein L27a OS=Mus musculus GN=Rpl27a PE=2 SV=5** | 246777.9 | 135424.7 | 1.82 |
| Q8R081 | **Heterogeneous nuclear ribonucleoprotein L OS=Mus musculus GN=Hnrnpl PE=1 SV=2** | 122230.0 | 66712.9 | 1.83 |
| Q62351 | **Transferrin receptor protein 1 OS=Mus musculus GN=Tfrc PE=1 SV=1** | 7845.1 | 4276.7 | 1.83 |
| Q91YR1 | **Twinfilin-1 OS=Mus musculus GN=Twf1 PE=1 SV=2** | 7161.5 | 3894.1 | 1.84 |
| P97825 | **Hematological and neurological expressed 1 protein OS=Mus musculus GN=Hn1 PE=1 SV=3** | 498423.4 | 268276.3 | 1.86 |
| Q9WUU7 | **Cathepsin Z OS=Mus musculus GN=Ctsz PE=2 SV=1** | 137474.0 | 73369.3 | 1.87 |
| Q8BG32 | **26S proteasome non-ATPase regulatory subunit 11 OS=Mus musculus GN=Psmd11 PE=1 SV=3** | 92543.5 | 49387.6 | 1.87 |
| P61961 | **Ubiquitin-fold modifier 1 OS=Mus musculus GN=Ufm1 PE=1 SV=1** | 75969.9 | 40514.7 | 1.88 |
| Q9WUK2 | **Eukaryotic translation initiation factor 4H OS=Mus musculus GN=Eif4h PE=1 SV=3** | 332496.9 | 177191.1 | 1.88 |
| Q07076 | **Annexin A7 OS=Mus musculus GN=Anxa7 PE=2 SV=2** | 36950.7 | 19676.8 | 1.88 |
| P09103 | **Protein disulfide-isomerase OS=Mus musculus GN=P4hb PE=1 SV=2** | 709953.7 | 377279.0 | 1.88 |
| Q8BK67 | **Protein RCC2 OS=Mus musculus GN=Rcc2 PE=1 SV=1** | 150298.1 | 79852.6 | 1.88 |
| P63028 | **Translationally-controlled tumor protein OS=Mus musculus GN=Tpt1 PE=1 SV=1** | 2351429.3 | 1247739.0 | 1.88 |
| Q6ZWV3 | **60S ribosomal protein L10 OS=Mus musculus GN=Rpl10 PE=1 SV=3** | 186215.8 | 98149.6 | 1.90 |

| | | | | |
|---|---|---|---|---|
| P26516 | **26S proteasome non-ATPase regulatory subunit 7 OS=Mus musculus GN=Psmd7 PE=1 SV=2** | 113226.7 | 58652.6 | 1.93 |
| O08810 | **116 kDa U5 small nuclear ribonucleoprotein component OS=Mus musculus GN=Eftud2 PE=2 SV=1** | 13013.0 | 6738.5 | 1.93 |
| P48428 | **Tubulin-specific chaperone A OS=Mus musculus GN=Tbca PE=2 SV=3** | 30410.2 | 15651.3 | 1.94 |
| Q08943 | **FACT complex subunit SSRP1 OS=Mus musculus GN=Ssrp1 PE=1 SV=2** | 52612.7 | 27006.1 | 1.95 |
| P62911 | **60S ribosomal protein L32 OS=Mus musculus GN=Rpl32 PE=1 SV=2** | 119215.2 | 60953.7 | 1.96 |
| Q78PY7 | **Staphylococcal nuclease domain-containing protein 1 OS=Mus musculus GN=Snd1 PE=1 SV=1** | 24591.9 | 12554.6 | 1.96 |
| P83917 | **Chromobox protein homolog 1 OS=Mus musculus GN=Cbx1 PE=1 SV=1** | 33129.7 | 16896.0 | 1.96 |
| P62830 | **60S ribosomal protein L23 OS=Mus musculus GN=Rpl23 PE=1 SV=1** | 225833.8 | 115031.2 | 1.96 |
| Q64010 | **Adapter molecule crk OS=Mus musculus GN=Crk PE=1 SV=1** | 34203.7 | 17139.6 | 2.00 |
| Q9EQ80 | **NIF3-like protein 1 OS=Mus musculus GN=Nif3l1 PE=1 SV=4** | 13878.1 | 6952.7 | 2.00 |
| Q99LX0 | **Protein deglycase DJ-1 OS=Mus musculus GN=Park7 PE=1 SV=1** | 494403.2 | 246490.1 | 2.01 |
| Q91VR5 | **ATP-dependent RNA helicase DDX1 OS=Mus musculus GN=Ddx1 PE=1 SV=1** | 6087.8 | 2977.7 | 2.04 |
| Q8CI51 | **PDZ and LIM domain protein 5 OS=Mus musculus GN=Pdlim5 PE=1 SV=4** | 2561.9 | 1249.6 | 2.05 |
| O88668 | **Protein CREG1 OS=Mus musculus GN=Creg1 PE=2 SV=1** | 72496.4 | 35327.6 | 2.05 |
| P13645 | **Contaminant (Keratin, type I cytoskeletal 10)** | 118219.8 | 57280.9 | 2.06 |
| P04264 | **Contaminant (Keratin, type II cytoskeletal 1)** | 616861.1 | 297231.6 | 2.08 |
| Q6P1F6 | **Serine/threonine-protein phosphatase 2A 55 kDa regulatory subunit B alpha isoform OS=Mus musculus GN=Ppp2r2a PE=1 SV=1** | 9705.8 | 4646.7 | 2.09 |
| P62075 | **Mitochondrial import inner membrane translocase subunit Tim13 OS=Mus musculus GN=Timm13 PE=1 SV=1** | 297131.4 | 141691.6 | 2.10 |
| P47968 | **Ribose-5-phosphate isomerase OS=Mus musculus GN=Rpia PE=2 SV=2** | 38648.3 | 18332.0 | 2.11 |
| P50431 | **Serine hydroxymethyltransferase, cytosolic OS=Mus musculus GN=Shmt1 PE=1 SV=3** | 29405.5 | 13891.1 | 2.12 |
| Q921I2 | **Kelch domain-containing protein 4 OS=Mus musculus GN=Klhdc4 PE=2 SV=2** | 33796.0 | 15555.1 | 2.17 |
| O89017 | **Legumain OS=Mus musculus GN=Lgmn PE=1 SV=1** | 19234.8 | 8708.9 | 2.21 |
| P62754 | **40S ribosomal protein S6 OS=Mus musculus GN=Rps6 PE=1 SV=1** | 337260.2 | 151284.3 | 2.23 |
| Q7M6Y3 | **Phosphatidylinositol-binding clathrin assembly protein OS=Mus musculus GN=Picalm PE=1 SV=1** | 10729.5 | 4753.4 | 2.26 |
| P97384 | **Annexin A11 OS=Mus musculus GN=Anxa11 PE=2 SV=2** | 12094.7 | 5316.1 | 2.28 |
| Q8BZA9 | **Fructose-2,6-bisphosphatase TIGAR OS=Mus musculus GN=Tigar PE=1 SV=1** | 5081.6 | 2174.9 | 2.34 |
| P48036 | **Annexin A5 OS=Mus musculus GN=Anxa5 PE=1 SV=1** | 334217.1 | 141593.6 | 2.36 |
| P10605 | **Cathepsin B OS=Mus musculus GN=Ctsb PE=1 SV=2** | 2091283.6 | 869948.8 | 2.40 |
| P13439 | **Uridine 5'-monophosphate synthase OS=Mus musculus GN=Umps PE=2 SV=3** | 30268.9 | 12508.7 | 2.42 |
| Q8VC28 | **Aldo-keto reductase family 1 member C13 OS=Mus musculus GN=Akr1c13 PE=1 SV=2** | 51113.8 | 20989.3 | 2.44 |
| O70435 | **Proteasome subunit alpha type-3 OS=Mus musculus GN=Psma3 PE=1 SV=3** | 360775.1 | 145649.1 | 2.48 |

| | | | | |
|---|---|---|---|---|
| P24472 | **Glutathione S-transferase A4 OS=Mus musculus GN=Gsta4 PE=1 SV=3** | 906179.3 | 356000.4 | 2.55 |
| O08807 | **Peroxiredoxin-4 OS=Mus musculus GN=Prdx4 PE=1 SV=1** | 51293.2 | 20028.7 | 2.56 |
| P25785 | **Metalloproteinase inhibitor 2 OS=Mus musculus GN=Timp2 PE=1 SV=2** | 20780.2 | 7937.8 | 2.62 |
| P07339 | **Contaminant (Cathepsin D)** | 113405.6 | 43062.9 | 2.63 |
| P45591 | **Cofilin-2 OS=Mus musculus GN=Cfl2 PE=1 SV=1** | 49402.5 | 18558.0 | 2.66 |
| P10852 | **4F2 cell-surface antigen heavy chain OS=Mus musculus GN=Slc3a2 PE=1 SV=1** | 14750.7 | 5463.8 | 2.70 |
| Q61207 | **Prosaposin OS=Mus musculus GN=Psap PE=1 SV=2** | 186532.6 | 66888.2 | 2.79 |
| Q11011 | **Puromycin-sensitive aminopeptidase OS=Mus musculus GN=Npepps PE=1 SV=2** | 85002.8 | 30477.0 | 2.79 |
| P35527 | **Contaminant (Keratin, type I cytoskeletal 9)** | 175688.4 | 62092.9 | 2.83 |
| Q6P542 | **ATP-binding cassette sub-family F member 1 OS=Mus musculus GN=Abcf1 PE=1 SV=1** | 2937.8 | 1035.9 | 2.84 |
| P23492 | **Purine nucleoside phosphorylase OS=Mus musculus GN=Pnp PE=1 SV=2** | 1154793.3 | 389536.8 | 2.96 |
| P24369 | **Peptidyl-prolyl cis-trans isomerase B OS=Mus musculus GN=Ppib PE=1 SV=2** | 199907.7 | 66628.7 | 3.00 |
| P12382 | **ATP-dependent 6-phosphofructokinase, liver type OS=Mus musculus GN=Pfkl PE=1 SV=4** | 5160.0 | 1596.9 | 3.23 |
| P28798 | **Granulins OS=Mus musculus GN=Grn PE=1 SV=2** | 86225.7 | 26445.7 | 3.26 |
| P17809 | **Solute carrier family 2, facilitated glucose transporter member 1 OS=Mus musculus GN=Slc2a1 PE=1 SV=4** | 4840.1 | 1479.2 | 3.27 |
| Q60598 | **Src substrate cortactin OS=Mus musculus GN=Cttn PE=1 SV=2** | 56381.7 | 16961.8 | 3.32 |
| Q9R0N0 | **Galactokinase OS=Mus musculus GN=Galk1 PE=2 SV=2** | 6521.6 | 1916.4 | 3.40 |
| P01887 | **Beta-2-microglobulin OS=Mus musculus GN=B2m PE=1 SV=2** | 127193.1 | 36423.5 | 3.49 |
| Q810D6 | **Glutamate-rich WD repeat-containing protein 1 OS=Mus musculus GN=Grwd1 PE=2 SV=2** | 15785.7 | 4455.0 | 3.54 |
| P51855 | **Glutathione synthetase OS=Mus musculus GN=Gss PE=2 SV=1** | 77787.2 | 21453.9 | 3.63 |
| Q3U0V1 | **Far upstream element-binding protein 2 OS=Mus musculus GN=Khsrp PE=1 SV=2** | 57768.5 | 15473.2 | 3.73 |
| P62082 | **40S ribosomal protein S7 OS=Mus musculus GN=Rps7 PE=2 SV=1** | 452059.4 | 115336.3 | 3.92 |
| Q8BFS6 | **Serine/threonine-protein phosphatase CPPED1 OS=Mus musculus GN=Cpped1 PE=2 SV=1** | 16738.2 | 4198.7 | 3.99 |
| P26043 | **Radixin OS=Mus musculus GN=Rdx PE=1 SV=3** | 12025.6 | 2802.0 | 4.29 |
| Q8BFU2 | **Histone H2A type 3 OS=Mus musculus GN=Hist3h2a PE=1 SV=3** | 325720.2 | 60469.9 | 5.39 |
| Q15843 | **Contaminant (NEDD8)** | 171850.5 | 31208.8 | 5.51 |
| Q60967 | **Bifunctional 3'-phosphoadenosine 5'-phosphosulfate synthase 1 OS=Mus musculus GN=Papss1 PE=1 SV=1** | 11519.5 | 1887.1 | 6.10 |
| Q61508 | **Extracellular matrix protein 1 OS=Mus musculus GN=Ecm1 PE=1 SV=2** | 45741.3 | 7460.4 | 6.13 |
| Q9WTX5 | **S-phase kinase-associated protein 1 OS=Mus musculus GN=Skp1 PE=1 SV=3** | 47081.8 | 4606.7 | 10.22 |
| Q07797 | **Galectin-3-binding protein OS=Mus musculus GN=Lgals3bp PE=1 SV=1** | 41826.1 | 0.0 | ***Specific in AF*** |
